# Industrial bees: The impact of apicultural intensification on local disease prevalence

**DOI:** 10.1111/1365-2664.13461

**Published:** 2019-07-16

**Authors:** Lewis J. Bartlett, Carly Rozins, Berry J. Brosi, Keith S. Delaplane, Jacobus C. de Roode, Andrew White, Lena Wilfert, Michael Boots

**Affiliations:** ^1^ Centre for Ecology and Conservation University of Exeter Penryn UK; ^2^ Department of Biology Emory University Atlanta Georgia; ^3^ Department of Integrative Biology University of California Berkeley California; ^4^ Department of Environmental Sciences Emory University Atlanta Georgia; ^5^ Department of Entomology University of Georgia Athens Georgia; ^6^ Department of Mathematics Heriot‐Watt University Edinburgh UK; ^7^ Institute of Evolutionary Ecology and Conservation Genomics University of Ulm Ulm Germany

**Keywords:** agriculture, apiculture, beekeeping, disease prevalence, infectious disease, intensification, mathematical model

## Abstract

1. It is generally thought that the intensification of farming will result in higher disease prevalences, although there is little specific modelling testing this idea. Focussing on honeybees, we build multi‐colony models to inform how “apicultural intensification” is predicted to impact honeybee pathogen epidemiology at the apiary scale.
2. We used both agent‐based and analytical models to show that three linked aspects of apicultural intensification (increased population sizes, changes in population network structure and increased between‐colony transmission) are unlikely to greatly increase disease prevalence in apiaries. Principally this is because even low‐intensity apiculture exhibits high disease prevalence.
3. The greatest impacts of apicultural intensification are found for diseases with relatively low R_0_ (basic reproduction number), however, such diseases cause little overall disease prevalence and, therefore, the impacts of intensification are minor. Furthermore, the smallest impacts of intensification are for diseases with high R_0_ values, which we argue are typical of important honeybee diseases.
4. *Policy Implications:* Our findings contradict the idea that apicultural intensification by crowding honeybee colonies in large, dense apiaries leads to notably higher disease prevalences for established honeybee pathogens. More broadly, our work demonstrates the need for informative models of all agricultural systems and management practices in order to understand the implications of management changes on diseases.

## 1 INTRODUCTION

Infectious diseases have significant impacts on agricultural sustainability (Brijnath, Butler, & McMichael, 2014) and profitability (James, 1981). A key question is how agricultural intensification and novel agricultural practices impact the emergence and epidemiology of infectious disease (Cressler, McLeod, Rozins, Hoogen, & Day, 2016; Gandon, Hochberg, Holt, & Day, 2013). It is generally assumed that intensification increases vulnerability to severe disease outbreaks (Jones et al., 2013; Kennedy et al., 2016; Mennerat, Nilsen, Ebert, & Skorping, 2010), but there is relatively little empirical data and, therefore, epidemiological theory is needed to address this problem (Atkins et al., 2013; Rozins & Day, 2016). Here, we build specific models of apiary‐level intensification in commercially farmed honeybees to examine the impact of industrial‐scale management practices on honeybee infectious disease prevalence.

Honeybee health and the apicultural industry are under threat from a variety of pressures (Ghazoul, 2005; vanEngelsdorp & Meixner, 2010), including parasites and pathogens (Budge et al., 2015; De la Rúa, Jaffé, Dall’Olio, Muñoz, & Serrano, 2009; Potts et al., 2010). There is a growing body of literature documenting the damage that emerging or re‐emerging diseases (Wilfert et al., 2016) are causing in apiculture (Jacques et al., 2017; Kielmanowicz et al., 2015) and native pollinators (Cohen, Quistberg, Philpott, & DeGrandi‐Hoffman, 2017; Fürst, McMahon, Osborne, Paxton, & Brown, 2014; Graystock, Blane, McFrederick, Goulson, & Hughes, 2016; Manley, Boots, & Wilfert, 2015; McMahon et al., 2015; McMahon, Wilfert, Paxton, & Brown, 2018). Evidence exists supporting a link between the risk of these diseases and specific apicultural practices (Giacobino et al., 2014; Mõtus, Raie, Orro, Chauzat, & Viltrop, 2016; Pacini et al., 2016). However, the evidence is geographically limited, lacking in mechanistic underpinning, or contradictory even within this small collection of studies. For example, Mõtus et al. (2016) report that larger apiaries show marginally higher incidence of ectoparasitic *Varroa* mites in Estonia, whilst Giacobino et al. (2014) did not find this association in a similar study in Argentina. It is, therefore, critical that we learn how different apicultural practices impact disease outcomes (Brosi, Delaplane, Boots, & de Roode, 2017). The need for an epidemiological framing of honeybee diseases has been frequently discussed (Brosi et al., 2017; Fries & Camazine, 2001) in both empirical (van Engelsdorp et al., 2013) and modelling (Becher, Osborne, Thorbek, Kennedy, & Grimm, 2013) studies, but we lack a modelling framework for disease ecology in honeybees at a scale larger than a single colony.

Honeybees are typically managed in apiaries, which are associated colonies placed together for beekeeping convenience at a single site. Pathogen dynamics at the apiary level are determined both by pathogen transmission within and between colonies. Intensification of apiculture changes apiary ecology in a number of ways, all potentially relevant to disease (Brosi et al., 2017). In particular, increasing the number of colonies and changing the arrangement of those colonies influences epidemiology through changes in both the size and network structure of the population. They both may also increase the rate at which transmission between colonies occurs via more frequent “drifting” of honeybees (Free, 1958; Neumann, Radloff, Pirk, & Hepburn, 2003). Drift is a key mechanism of between‐colony pathogen transmission (Goodwin, Perry, & Houten, 1994; Roetschi, Berthoud, Kuhn, & Imdorf, 2008) and has been invoked as an explanatory mechanism accounting for higher parasite prevalences in larger apiaries (Mõtus et al., 2016).

The intensification of agricultural systems generally means larger, denser population sizes and greater pathogen transmissibility at local (within a population, such as a farm) and landscape (between populations, such as neighbouring farms) scales. To understand these effects in honeybees we build multi‐colony models to examine how apicultural intensification is predicted to impact honeybee pathogen epidemiology. We examine the epidemiological consequences of increasing the number of colonies within an apiary, changing colony configurations, and increasing between‐colony pathogen transmission.

## 2 MATERIALS AND METHODS

We combine mathematical models and agent‐based model (ABM) simulations to make predictions on how intensification affects disease risk, spread and endemic prevalence within an apiary. The key to our approach is that we capture pathogen transmission both within and between colonies.

**Figure 1:**
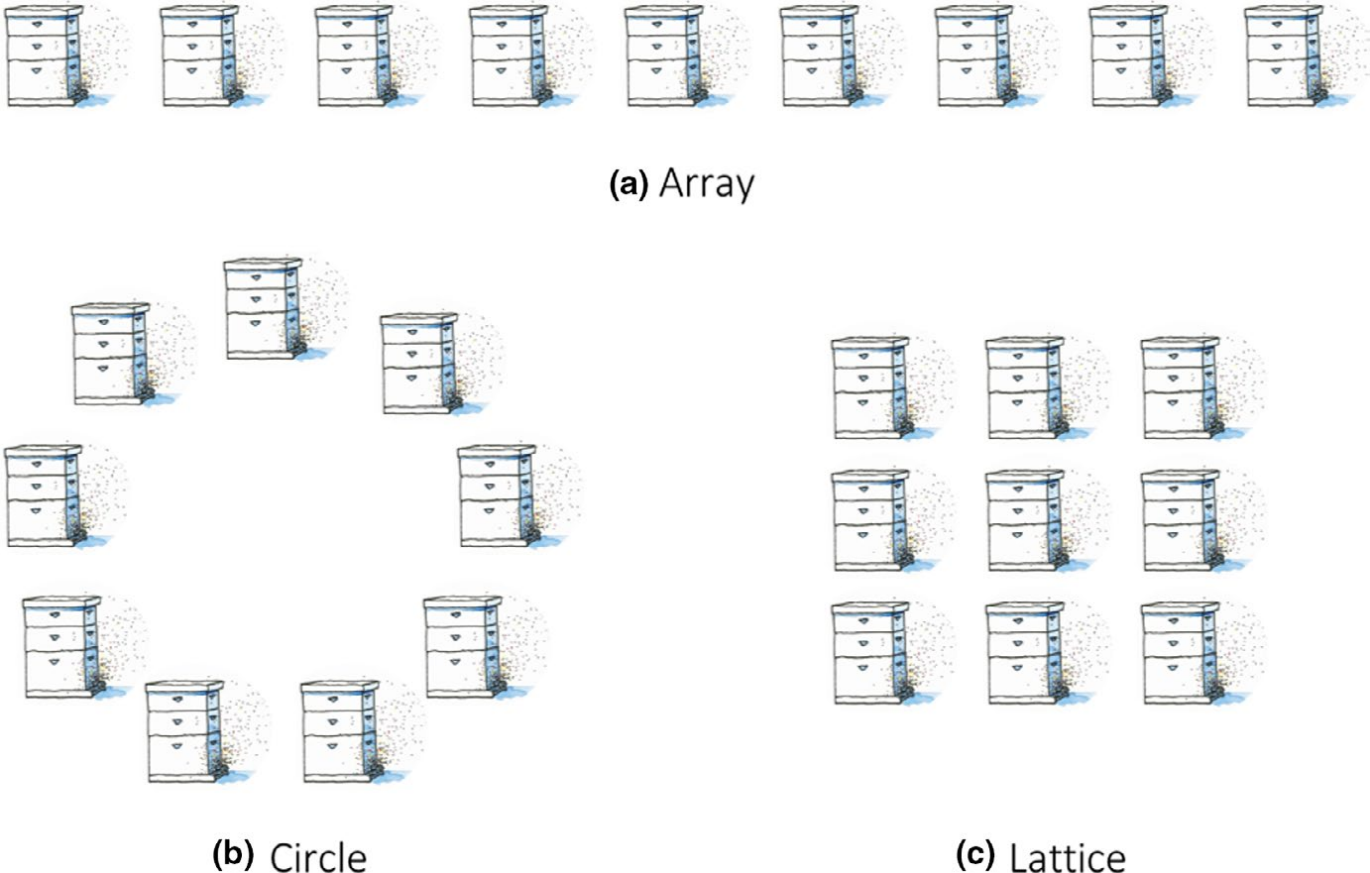
Colony configurations, demonstrated for apiaries with nine colonies

**Figure 2:**
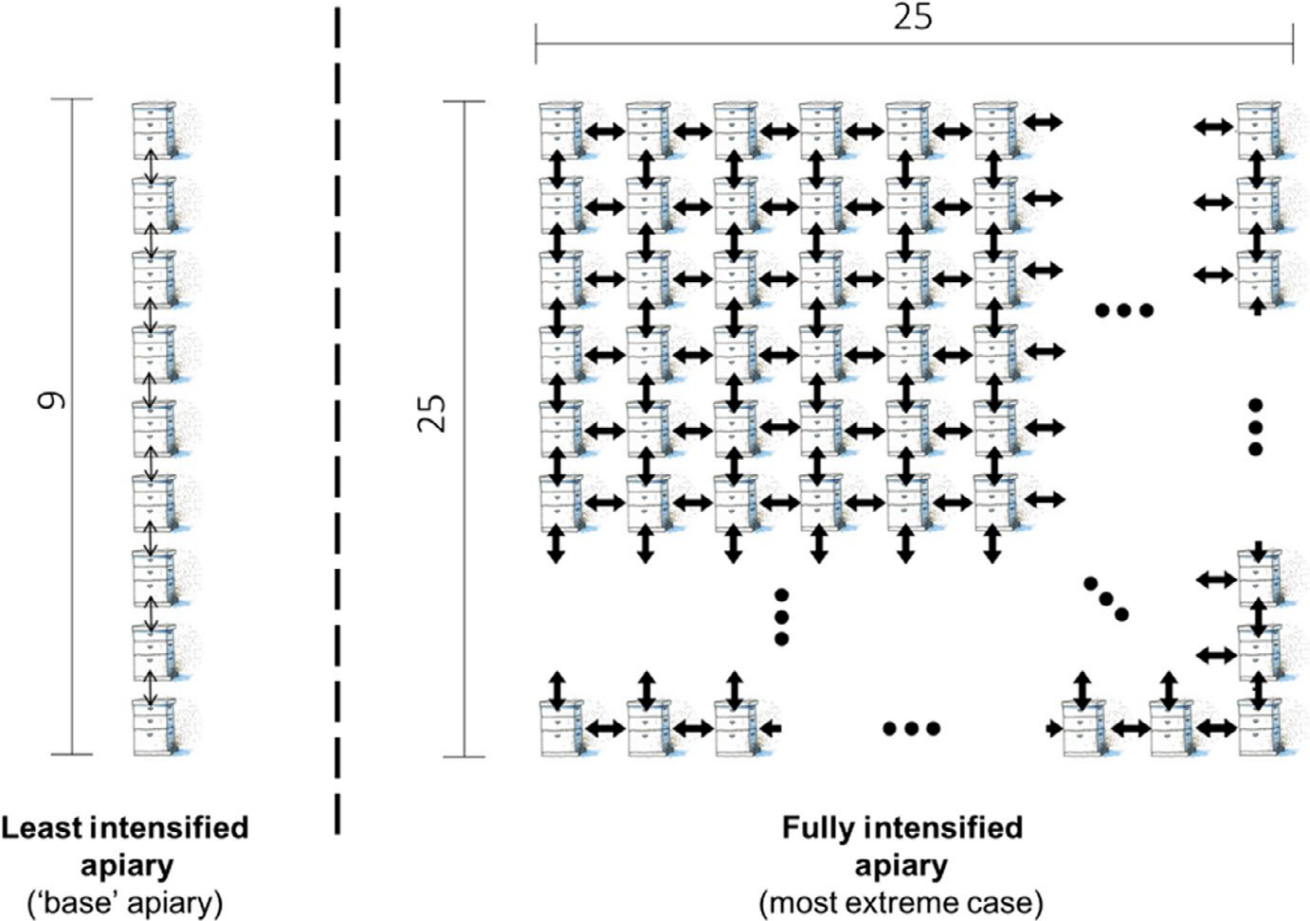
Illustrative schematic of the “intensification” treatment as it is used in parts of this manuscript. We show the apiary used to estimate “base R_0_” (left) compared to the intensified apiary (right) reflecting an increase in number of colonies from 9 to 225, a change from an array to a lattice, and a 10‐fold increase in movement of honeybees between colonies (illustrated using arrow weight) from a likelihood of 0.015 per bee per day to 0.15. Note that for the intensified apiary, not all 225 colonies are shown, with missing colonies denoted by ellipses (...)

We generalize colony arrangements to three unique configurations drawn from experience, classic apicultural literature (Jay, 1966) and current experimental work (Dynes, Berry, Delaplane, Brosi, & de Roode, 2019): array, circular and lattice (Figure 1). We restrict between‐colony pathogen transmission to nearest neighbours (see discussion), those in closest proximity to each other (connected by an arrow in Figure 2). Between‐colony transmission is always assumed to be at a lower rate than within colony transmission. The mathematical model allows us to obtain tractable analytical results while the ABM simulations allow us to model disease at the level of the individual bee and consider stochastic effects.

We first derive a compartmental SI (Susceptible, Infected) model for pathogen transmission within an apiary. The model treats each colony as an individual population and allows for within‐colony as well as between‐colony transmission (for nearest neighbours). Within a colony, honeybees are either susceptible to infection or infected (and infectious). We denote the number of susceptible honeybees in colony *i* at time *t* as *S_i_*(*t*). Likewise, we denote the number of honeybees in colony *i* infected with the pathogen at time *t* as *I_i_*(*t*). Susceptible honeybees in colony *i* become infected at rate *β_ij_* following contact with an infected bee that resides in colony *j*. We assume that honeybees do not recover from infection. Honeybees are born at rate *ϕ*, have a natural mortality rate of *m* and an additional mortality rate of *v* if infected. The following *2n* differential Equations, (1), model disease transmission within and between *n* colonies in an apiary.

(1) $$\begin{array}{c} \frac{\text{d}S_{i}}{\text{d}t}=-\sum_{j=1}^{n}\beta_{\mathrm{ij}}S_{i}I_{j}-mS_{i}+\phi \\ \frac{\text{d}I_{i}}{\text{d}t}=\sum_{j=1}^{n}\beta_{\mathrm{ij}}S_{i}I_{j}-\left(m+v\right)I_{i} \end{array}$$

The matrix *β* = [*β_ij_*] will depend on the colony arrangement (see Figure 1; and S.I. Section 1). The transmission rate between a susceptible and infected honeybee within the colony is *a*, and transmission between neighbouring colonies is *b*. For example, for a nine‐colony apiary, the transmission matrices for an array, circular and lattice configured apiary (respectively) are as follows:

$$\left\lceil \begin{array}{ccccccccc} a & b & 0 & 0 & 0 & 0 & 0 & 0 & 0\\ b & a & b & 0 & 0 & 0 & 0 & 0 & 0\\ 0 & b & a & b & 0 & 0 & 0 & 0 & 0\\ 0 & 0 & b & a & b & 0 & 0 & 0 & 0\\ 0 & 0 & 0 & b & a & b & 0 & 0 & 0\\ 0 & 0 & 0 & 0 & b & a & b & 0 & 0\\ 0 & 0 & 0 & 0 & 0 & b & a & b & 0\\ 0 & 0 & 0 & 0 & 0 & 0 & b & a & b\\ 0 & 0 & 0 & 0 & 0 & 0 & 0 & b & a \end{array}\right\rceil ,\;\left\lceil \begin{array}{ccccccccc} a & b & 0 & 0 & 0 & 0 & 0 & 0 & b\\ b & a & b & 0 & 0 & 0 & 0 & 0 & 0\\ 0 & b & a & b & 0 & 0 & 0 & 0 & 0\\ 0 & 0 & b & a & b & 0 & 0 & 0 & 0\\ 0 & 0 & 0 & b & a & b & 0 & 0 & 0\\ 0 & 0 & 0 & 0 & b & a & b & 0 & 0\\ 0 & 0 & 0 & 0 & 0 & b & a & b & 0\\ 0 & 0 & 0 & 0 & 0 & 0 & b & a & b\\ b & 0 & 0 & 0 & 0 & 0 & 0 & b & a \end{array}\right\rceil ,\;\left\lceil \begin{array}{ccccccccc} a & b & 0 & b & 0 & 0 & 0 & 0 & 0\\ b & a & b & 0 & b & 0 & 0 & 0 & 0\\ 0 & b & a & 0 & 0 & b & 0 & 0 & 0\\ b & 0 & 0 & a & b & 0 & b & 0 & 0\\ 0 & b & 0 & b & a & b & 0 & b & 0\\ 0 & 0 & b & 0 & b & a & 0 & 0 & b\\ 0 & 0 & 0 & b & 0 & 0 & a & b & 0\\ 0 & 0 & 0 & 0 & b & 0 & b & a & b\\ 0 & 0 & 0 & 0 & 0 & b & 0 & b & a \end{array}\right\rceil$$

The corresponding network structures for the above transmission matrices can be seen in Figure S1. We assume that honeybees are much more likely to become infected by a honeybee that resides within its home colony than by a honeybee from a neighbouring colony (i.e. *a *» *b*). Note that for each apiary configuration to be possible and unique, the number of colonies (*n*) must be a perfect square, *n = L^2^* where *L* ≥ 3 (see Figure 1). Therefore, the minimum number of colonies per apiary is nine, which has been observed to be the mean size of a hobbyist or small beekeeping operation (Mõtus et al., 2016; Pocol, Marghitas, & Popa, 2012).

We complement our mathematical model (1) with the ABM; our ABM simulates pathogen spread, through individual bee movements, across an apiary. Apiaries are differentiated by the same characteristics as in the mathematical model; a description of the ABM is available in the S.I. (Section 2) and the model is publicly available (see S.I.). We use the ABM to simulate disease dynamics for both different pathogen phenotypes (varying both pathogen virulence and transmissibility) and different apiary ecologies (varied as previously described in the number of colonies per apiary, layout and likelihood of bees moving between colonies) (S.I. Figures S3 and S4); we compare the ABM to the analytical model and use it to test assumptions made elsewhere in the study (Figure 4a, S.I. Figure S6).

We can understand the dynamics presented by our models by focussing on the basic reproduction number, R_0_. R_0_ is a fundamental concept in infectious disease ecology, defined as the average number of secondary infections caused by one infectious individual in an otherwise entirely susceptible population (Anderson & May, 1992). We derive R_0_ expressions, using model (1), for each of the apiary configurations. R_0_ derivations using model (1) allow us to characterize the relationship between R_0_ and pathogen prevalence, defined as the proportion of honeybees within an apiary that are infected at the endemic equilibrium. The R_0_ expressions for apiaries with *n *>* *1 colonies were calculated using the next generation method (van den Driessche & Watmough, 2002), (see S.I. Section 1).

(2a) $${\text{R}}_{0\text{Array}}=\frac{\phi }{m\left(m+v\right)}\left(a-2b\cos\frac{n\pi }{n+1}\right)$$

(2b) $${\text{R}}_{0\text{Circle}}=\frac{\phi }{m\left(m+v\right)}\left(a+2b\right)$$

(2c) $${\text{R}}_{0\text{Lattice}}=\frac{\phi }{m\left(m+v\right)}\left(a-4b\cos\frac{\sqrt{n}\pi }{\sqrt{n}+1}\right)$$

For the ABM we estimate R_0_ values for particular parameter combinations by treating simulation outputs as ideal empirical data (Keeling & Rohani, 2008) and track the number of infections following the index case. The term “base R_0_” is used throughout the remainder of this paper and refers to a value of R_0_ for a specific pathogen phenotype in a least intensified apiary, an array with nine colonies (see Figure 2). We determine how intensification affects R_0_ by separating R_0_ into a “base R_0_” and an “additional R_0_”. The term “additional R_0_” refers to the observed difference in R_0_ for a given pathogen phenotype when comparing a “lower intensity” apiary to a “high intensity” one (Figure 2).

An extreme, but plausible, example of intensification is used for these comparisons. Specifically, an increase in colonies per apiary from 9 to 225 colonies, a change to a lattice configuration and a 10‐fold increase in between‐colony infection (0.015–0.15 per bee per day), demonstrated in Figure 2. The difference in the R_0_ before and after intensification is how we estimate “additional R_0_”. This permits the interaction (nonadditive) effects of our three aspects of intensification. The “additional R_0_” can then be used in combination with the analytically derived relationship between R_0_ and prevalence (see model (1) and Equations (2a–c)) to characterize how intensification affects disease prevalence. We focus on disease prevalence as both models show rapid pathogen spread across apiaries, such that infection prevalence at the endemic equilibrium was the major result differentiating modelling scenarios (S.I. Figures S4 and S5).

## 3 RESULTS

Our main results constitute three main characterizations of this system: the relationship between R_0_ and pathogen prevalence; the effects of intensification on R_0_; and by combination of these relationships, the effect of intensification on pathogen prevalence. The relationship between R_0_ and pathogen prevalence is principally derived from the analytical model (presented first in these results) but is confirmed to broadly agree with the agent‐based model (presented second). The relationship between intensification and R_0_ is principally derived from the ABM, presented second, but is partly explored in the analytical model presented first. The critical overall result is the combination of these relationships, presented last and visualized in Figure 5, demonstrating how intensification impacts disease prevalence. Detailed derivation, exploration and testing of both models is detailed in the Supplementary Information.

**Figure 3:**
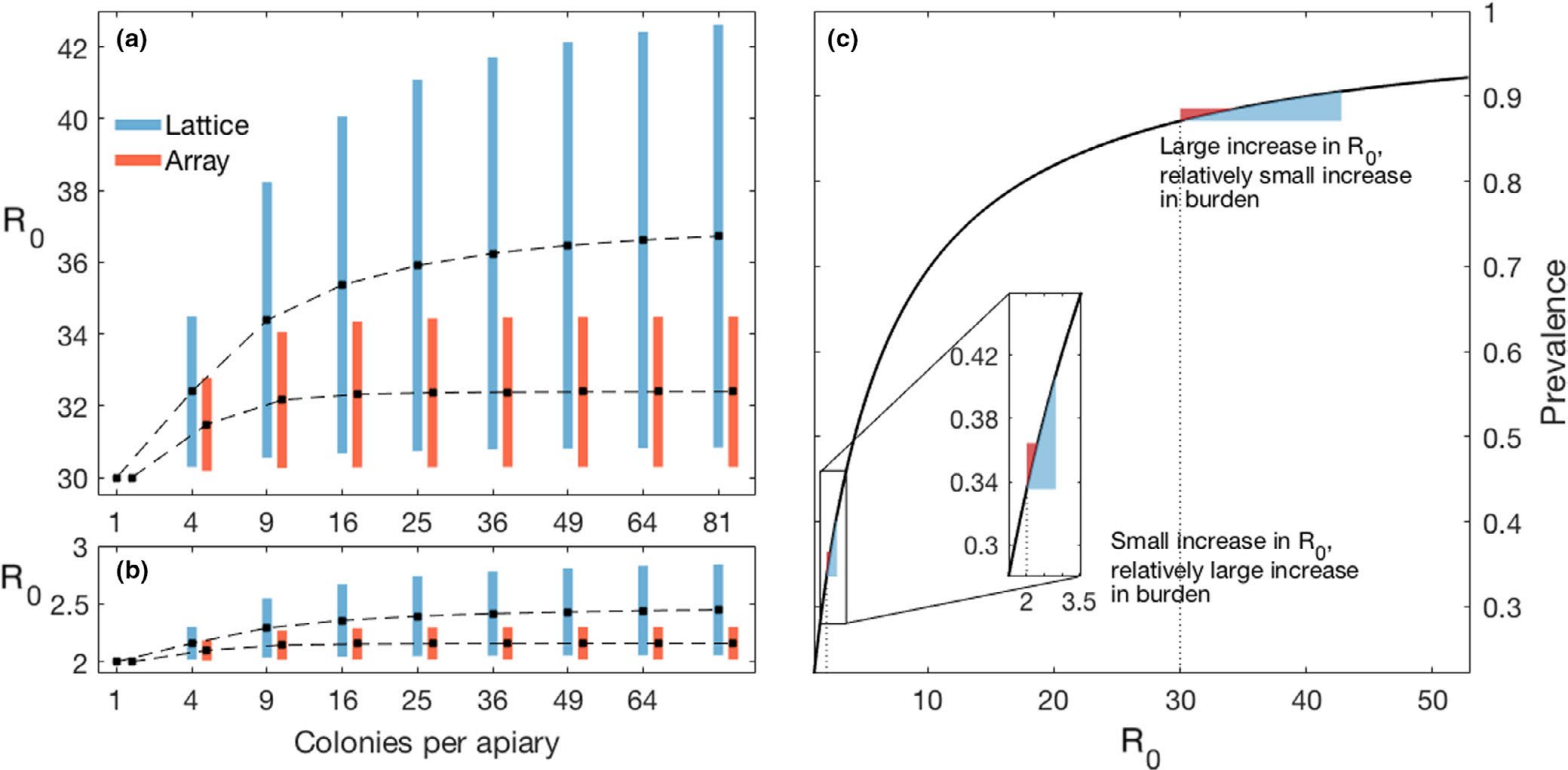
Relationships between number of colonies, R_0_, and prevalence from model (1). Figure 3a and 3b demonstrate that the effect on R_0_ for different degrees of intensification rapidly asymptotes, justifying our “single intensification” treatment (Figure 2). Figure 3c defines the relationship between R_0_ and prevalence, the shape of which critically determines our main result (see Figure 5). Technical description: (a) When R_0_ = 30 for a single‐colony apiary, the addition of colonies yields a maximum increase in R_0_ of 12.7 for the lattice and 4.5 for the array. (b) When R_0_ = 2 for a single colony, there is a maximum increase in R_0_ of 0.85 for the lattice and 0.29 for the array, when colonies are added. Recall that the R_0_ for the circle is independent of *n* (see (2b)), and hence absent from the figure. Parameter values are set to: *v* = 0.1, *m* = 0.0272, ϕ = 1,600 and in a) *a + b* = 4.32485 × 10^‐6^ and in b) *a + b* = 6.48725 × 10^‐5^. The transmissibility is what affects base R_0_. Black dots are values where between‐colony transmission is held at 10% of total transmission, with the bottom and top of the bars representing 1% and 20% of the total transmission being between hives, “b”, respectively. (c) The relationship between R_0_ and disease prevalence. The range of R_0_ values is generated by varying the overall transmission rate (i.e. *a + b*) from 2.143 × 10^‐6^ to 1.178 × 10^‐4^ as reported by Roberts and Hughes (2015) for *Nosema ceranae*

Both model (1) and the ABM simulations show that, for a given number of colonies per apiary, R_0_ is always greatest for the lattice arrangement—the most highly connected configuration. As the number of colonies per apiary increases (increasing *n*), the values of R_0_ in both the array and lattice configurations increase (Figure 3a,b), while the R_0_ for the circular configuration remains unchanged (see R_0_ equations). The increase in R_0_ from the addition of colonies asymptotes quickly due to convergence in the mean number of neighbours across the apiary; this is also why the R_0_ for the circular apiary is independent of number of colonies as the number of neighbours per colony remains two. This explains why R_0_ for an array arrangement approaches the R_0_ value for a circular arrangement as the number of colonies increases.

If R_0_ > 1, the pathogen will rapidly invade (see S.I. Section 1, Figure S5) and each colony will reach a stable population size and infection prevalence, called the endemic equilibrium (See S.I. Section 1). Mathematically the disease prevalence at equilibrium for colony *j* is I_j_*/(I_j_*+S_j_*), where S_j_* is the number of susceptible honeybees and I_j_* is the number of infectious honeybees in colony *j* at equilibrium. The endemic equilibrium for the circular configuration model can be solved explicitly (see S.I. Section 1). Due to symmetry, all colonies within the circular apiary have disease prevalence at the endemic equilibrium of:

$$\frac{\phi \left(a+2b\right)-m\left(m+v\right)}{\phi \left(a+2b\right)+v\left(m+v\right)}$$

We can approximate the endemic equilibrium for the lattice and array configured models using perturbation theory, assuming 0 < *b* « 1 (See S.I. Section 1). The approximate disease prevalence in colony *j* at equilibrium for a colony in the array or lattice configurations is:

$$\frac{\phi a^{2}+lbm\left(m+v\right)}{\phi a^{2}+a{\left(m+v\right)}^{2}-blv\left(m+v\right)}$$

where *l* is the number of neighbours that colony *j* has. For any given set of parameters, we can, therefore, formulate both R_0_ and prevalence, allowing us to characterize the relationship shown in Figure 3c.

**Figure 4:**
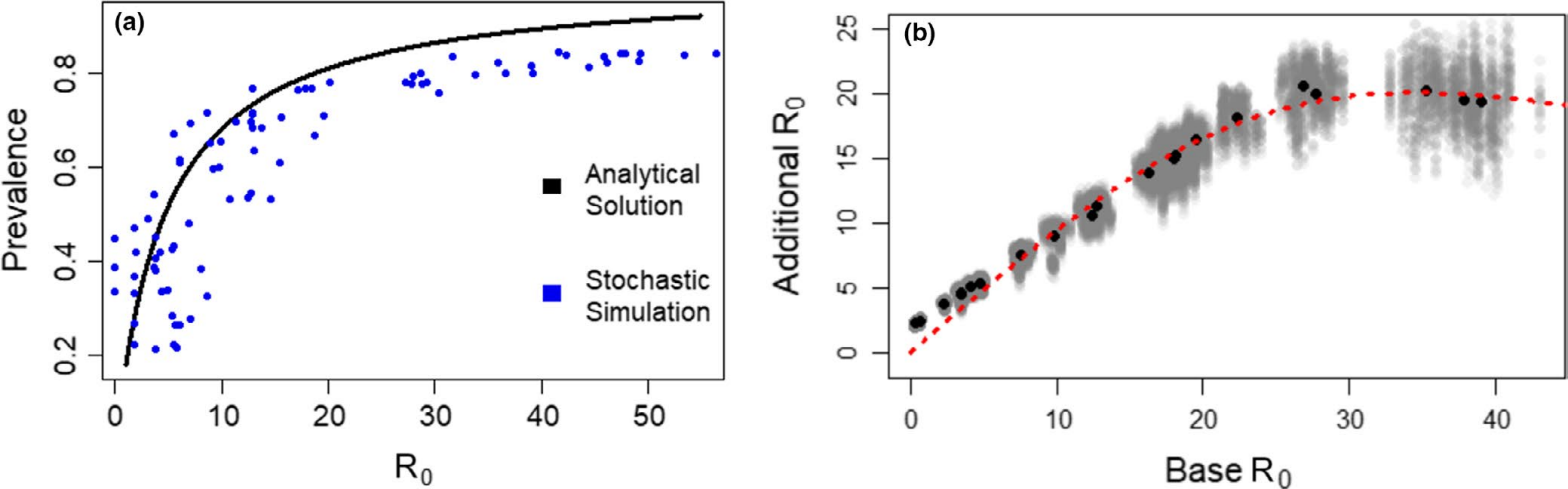
Results from the ABM. Figure 4a demonstrates the agreement between the ABM and analytical model; Figure 4b presents the critical relationship estimated from the ABM relating base R_0_ to the increase in R_0_ following intensification (see Figure 2), the shape of which critically determines our main result (see Figure 5). Technical description: (a) shows agreement between the stochastic simulations (ABM) and analytical model (Figure 3c); using the following equivalent model parameterization to that for Figure 3c: Circular configuration, *n* = 9, *M* = 58,200, Φ = 1,600, 5 × 10^‐6^ ≤ *β* ≤ 1 × 10^‐4^, *ν* = 0.1, *ρ* = 0.1 (see S.I. Section 2). (b) Examines how an extreme example of intensification (see Figure 2) alters R_0_ across a range of different “base R_0_” values determined by pathogen phenotype using the ABM. Grey points represent individual simulation comparisons, black points represent mean values. Base R_0_ values are unevenly distributed across the range due to R_0_ being an emergent property of the system in both plot panels. We derive a nonlinear relationship between “base R_0_” and “additional R_0_” for panel b, corresponding to Figure 2 (see Figure 2 for panel b parameterization, otherwise as listed for a, plotted as a dashed red line. Variation within clusters is a result of the stochastic simulations

We show analytically, and in the ABM (S.I. Section 3) that intensification in the form of an increase in colonies or an increase in movement between colonies increases R_0_ (Figure 3a, b). Figure 4b shows the additional R_0_ caused by our most extreme plausible changes in apiary management. The change in R_0_ caused by increasing apiary size rapidly asymptotes (Figure 3a, b).

The effect of intensification is dependent on the base R_0_—for small base R_0_, intensification causes little additional R_0_, but at intermediate or high base R_0_, intensification leads to large additional R_0_ (Figure 4b). While the increase in R_0_ is largest for an already large base R_0_, this relationship saturates and the relative increase in R_0_ for a given base R_0_ stays relatively constant for large base R_0_ values. The relationship shows a strong nonlinearity when examining all three aspects of intensification in combination.

By understanding the effect of intensification on R_0_ (Figure 4b) and by characterizing the relationship between R_0_ and disease prevalence (Figures 3c and 3a), we can show how intensification impacts disease prevalences. We approximate the nonlinear relationship between “base R_0_” (pathogen phenotype) and the “additional R_0_” (effect of intensification) in Figure 4b. We use a bootstrapping approach to create 1,000 subsamples (subsample size = 10% of full sample with replacement) of our combined approach. Each subsample is used to generate a nonlinear model of the form *y = ax*/(*b + x^c^*), where *y* is “additional R_0_” and *x* is “base R_0_”, using a nonlinear least squares approach in R (v 3.3.1). The relationship generated using the full sample is plotted in Figure 4b.

**Figure 5:**
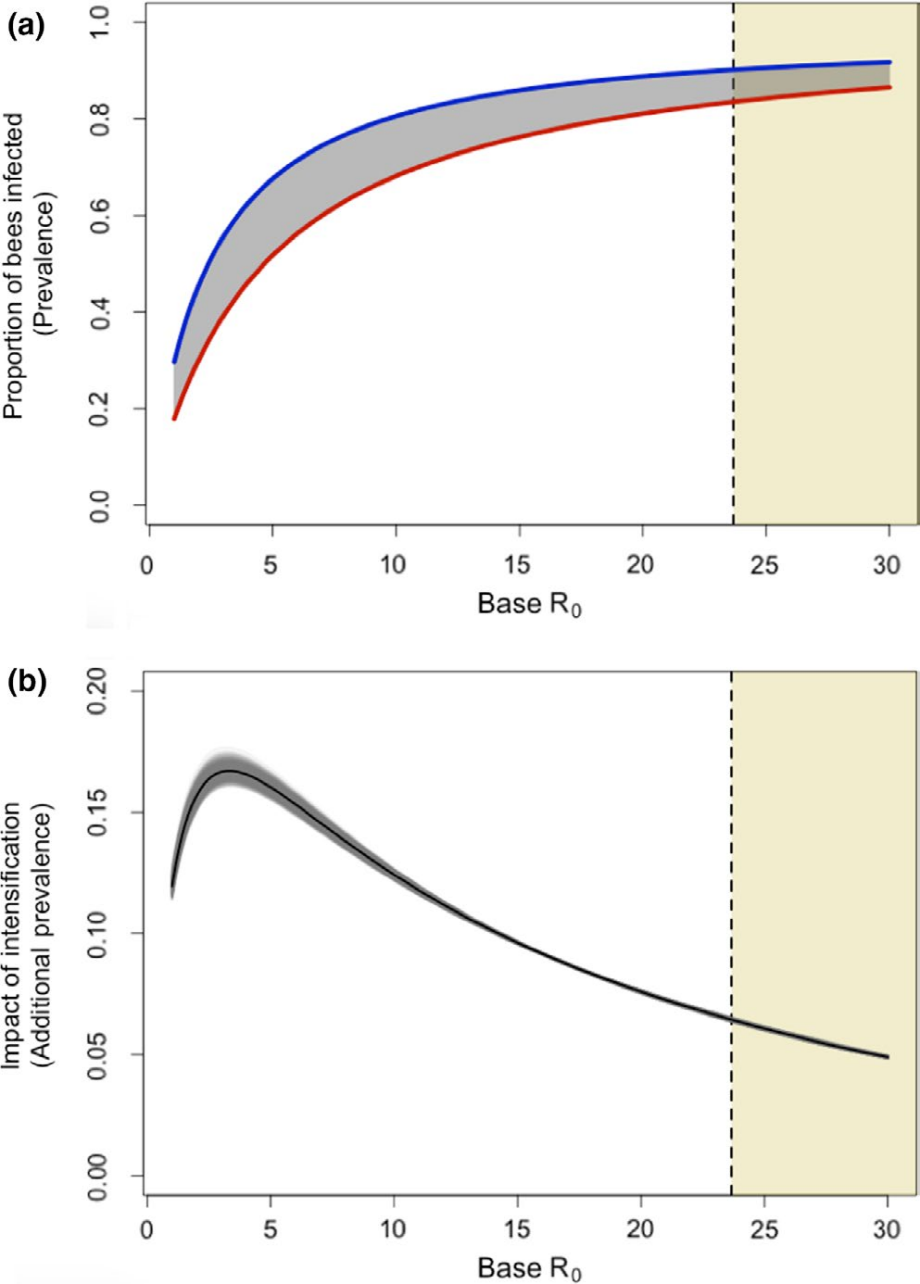
Depictions of our critical finding characterizing the maximum (peak), and likely (shaded region), increases in prevalence of a pathogen following local intensification of apiculture. High prevalence even in “low intensity” (see Figure 2) systems yields little opportunity for large increases in prevalence. Panel (a) shows the proportion of bees infected (prevalence) in non‐intensified apiaries (lower red line) compared to intensified apiaries (upper blue line), take from the mean values derived in Figure 4b and the relationship shown in Figure 3c. The shaded grey area between these curves is the additional prevalence caused by intensification—the “impact of intensification”. This is plotted in panel (b) where the black line represents the mean relationship, and the grey lines represent 1,000 bootstrapped samples. The vertical dashed line and yellow‐shaded region of the graphs to the right of the dashed line show a lowest estimated value of R_0_ for *Nosema ceranae*. Figures start at R_0_ = 1.0008

We combine this relationship characterizing how base R_0_ affects intensified additional R_0_ (Figure 4b) with the derived relationship between R_0_ and pathogen prevalence shown in Figure 3c, allowing us to predict how intensification impacts prevalences (Figure 5). Figure 5a shows the proportion of bees infected by a given (base R_0_) pathogen for the two apiaries in Figure 2. The difference in disease prevalence between these lines is the impact of intensification and is plotted in Figure 5b. Figure 5b shows a distinctly peaked relationship between base R_0_ and the impact of intensification, with the impact of intensification peaking around base R_0_ = 3.3, and then rapidly declining. Even at its peak, the effect of intensification (which is as extreme as plausible), leads to an additional ~18% of bees infected at disease equilibrium. We present Figure 5 as the most important graphic for understanding the overall conclusions of this study, as the apparent “small” shift in R_0_ required to double prevalence (Figures 3c and 4a) is actually very difficult to achieve for low R_0_ pathogens (see Figures 3b and 4b), resulting in the “maximum plausible” change shown by the peak in Figure 5b (~18.5%).

We contextualize these results by calculating an estimate of the lower‐bound of R_0_ value for a honeybee pathogen (see highlighted regions in Figure 5). We identified this region based on empirical data for the microsporidian pathogen *Nosema ceranae*; this was the only pathogen for which experimentally derived transmission rates as well as robust information on mortality due to infection is available (Martín‐Hernández et al., 2011; Paxton, Klee, Korpela, & Fries, 2007; Roberts & Hughes, 2015). To estimate the plausible R_0_ boundary in our model for this pathogen, we parameterized our mathematical model using the lowest empirically supported transmission value with the highest supported additional mortality, and fixed movement of honeybees between colonies at its lowest supported natural rate (Currie & Jay, 1991). We then calculated the R_0_ for a circular apiary due to its scale independence.

## 4 DISCUSSION

Our results present a counterintuitive picture of apicultural intensification and its consequences on disease prevalence within apiaries. Even in their most plausibly extreme cases, changes in the number of colonies, their spatial arrangement and transmission rates between colonies (reflecting management intensification (Brosi et al., 2017)) had only a small effect on the severity of disease at the apiary level for pathogens of interest. Apicultural intensification leads to large gains in R_0_ when R_0_ is initially high and small gains in R_0_ when R_0_ is initially low (Figure 4b). However, increases in R_0_ cause large increases in prevalence only when R_0_ is initially low (Figures 3c and 4a). Pathogens with a base R_0_ ≈ 3 benefit most from intensification in terms of increased prevalence (Figure 5); As discussed below, we argue that there is likely to be a high base R_0_ in important honeybee diseases and, therefore, our models suggest that there is likely to be little effect of apiary‐scale intensification on disease prevalences. However, if a pathogen emerges with a relatively low R_0_, our model does indicate that extreme intensification could lead to a significant increase in prevalence of approximately 18.5%. Therefore, if intensification increases the risk of novel pathogen emergence, then these newly emerged pathogens would benefit from intensification, as it would significantly increase their disease prevalence, relative to the pre‐intensified apiary.

Our models most closely resemble the ecology of a directly transmitted microparasite able to infect individual honeybees at any life stage, conceptually similar to the microsporidian pathogens *Nosema* spp. (Fantham & Porter, 1912). *Nosema* is a major concern to beekeepers world‐wide (Higes et al., 2008, 2009; Paxton, 2010), and has a minimum estimated base R_0_ of 23 (Figure 5) when modelled here. We found that apicultural intensification, in the context of a pathogen with an initial R_0_ of 23, leads to a maximum 6.6% increase in disease prevalence. Our models predicted disease prevalences of up to 90% (Figure 3, Figure 5; S.I. Section 3), which while high, are empirically supported for the honeybee system (Higes et al., 2008; Kielmanowicz et al., 2015), and feature in other modelling studies that use similar transmission parameters to ours (Betti, Wahl, & Zamir, 2014). *Nosema* was the only pathogen for which there are direct empirical studies characterizing its transmissibility, however, other honeybee pathogens such as deformed wing virus are also well studied. While estimating an R_0_ for DWV is difficult due to active management by beekeepers, maximum reported prevalences that may be indicative of its true “unmanaged” R_0_ are high, for example 73% in Natsopoulou et al. (2017), 80% in Budge et al. (2015) and 100% in Stamets et al. (2018). These high prevalences are consistent with high R_0_ values (Figures 3c and 4a, and S.I. (Section 3)).

We additionally explored the behaviour of a more specific model, using an age‐structured approach to infection dynamics, where only larvae are vulnerable to infection and develop into infectious adults with a high pathogen‐associated mortality (as might be appropriate for pathogens such as the acute paralysis virus complex (Martin, 2001)), presented in the S.I. (Section 3). Convergence to equilibrium happens more slowly than the main model presented here, but still occurs quickly (within a single beekeeping season; see S.I. 3 Figure S7). However adult‐bee infection prevalence is far lower than seen in our SI model (S.I. Figure S7)—this is in agreement with observations of lower prevalence of paralysis viruses (Budge et al., 2015). Notably, the endemic equilibrium prevalence increases only by small magnitudes as movement between colonies or apiary sizes are drastically increased (S.I. Figure S7), in agreement with our main general result. This equivalence in behaviour between different models reflecting large disparities in infection mechanics and different endemic prevalences demonstrates that these results are likely generalizable to many honeybee pathogens.

We find rapid spread of a given pathogen across an apiary, which quickly reaches endemic equilibrium (S.I. Figures S4–S6). While pathogens with a higher R_0_ reach this equilibrium more quickly, there is universally rapid spread. Given this result, we mainly focussed on the disease prevalence experienced at endemic equilibrium. Despite assuming transmission only to nearest neighbours, pathogen spread occurs rapidly, and the nearest neighbour assumption alters this very little when removed or relaxed (see S.I. Figure S6). The rate at which epidemics are established in our model is also in agreement with other honeybee pathogen models. For example, Jatulan, Rabajante, Banaay, Fajardo, and Jose (2015) show that a single infectious adult causes an American Foulbrood (*Paenibacillus larvae*) epidemic that peaks within 50 days. Whilst they do not explicitly find an R_0_ for *P. larvae*, the short timescales characterizing their epidemics are in line with ours (S.I. Section 3), suggesting high R_0_ values and that their model would behave similarly to ours at an apiary scale.

Our intercolony transmission can be understood to capture multiple processes arriving from beekeeper management such as brood transplantation or reduced distance between colonies (Brosi et al., 2017) as well as recognized transmission routes such as honeybee drift (Jay, 1965). Our approach was informed by studies which have focussed on how changes in the number of colonies and apiary configurations (Jay, 1966, 1968) alter drift (Dynes et al., 2017). Links between drift‐mediated pathogen transmission and colony numbers have been documented for a variety of pathogens (Seeley & Smith, 2015)—including brood specialized and non‐specialized, micro‐ and macro‐parasites (Belloy et al., 2007; Budge et al., 2010; Dynes et al., 2017; Nolan & Delaplane, 2017). Larger numbers of colonies per apiary are a driver of higher drift (Currie & Jay, 1991), as are changes in apiary arrangement (Dynes et al., 2019; Jay, 1966). While beekeepers typically maintain equal distances between their colonies regardless of how many colonies are in the apiary (such that larger apiaries have a bigger area footprint), our approach of increasing between‐colony transmission in larger apiaries would also capture any additional transmission from spatial crowding.

Two clear candidates for future development of this model include seasonality and demography, which are closely linked. Honeybee demography within a colony influences epidemiology (Betti, Wahl, & Zamir, 2016) due in part to the temporal polyethism of task allocation influencing exposure and immunity (Calderone & Page, 1996), as well as the flexible ability of honeybees to regain immune function when they revert roles (Amdam et al., 2005; Robinson, Page, Strambi, & Strambi, 1992). However, patterns in how age and immunosenescence in honeybees relates to survival and infectiousness remain complicated (Roberts & Hughes, 2014). Analytically tractable models accounting for the role of this complex demography in understanding stress in a colony have only recently been developed (Booton, Iwasa, Marshall, & Childs, 2017), and extending these models to incorporate diseases at the apiary scale is challenging. However, notable phenomena worth pursuing include: the role of male bees, which are known to be more easily infected, more infectious and more likely to drift between colonies (Currie & Jay, 1991; Roberts & Hughes, 2015); as well as the role of robbing—where honeybees invade other colonies to steal food (Fries & Camazine, 2001; Lindström, Korpela, & Fries, 2008).

At broader scales, overstocking of colonies may lead to resource limitation and consequently impaired immune function (Al‐Ghamdi, Adgaba, Getachew, & Tadesse, 2016; Pasquale et al., 2013). These effects are important for a broader understanding of honeybee epidemiology, but should be separated from the within‐apiary processes studied here. Additionally, most honeybee infectious diseases are caused by multi‐host pathogens shared with other wild bees (Fürst et al., 2014; Manley et al., 2015; McMahon et al., 2015, 2018). Honeybee colony density across a landscape, therefore, has implications for wild pollinator health (Cohen et al., 2017; Graystock et al., 2016), however, our results suggest that increased stocking of honeybees may have smaller impacts on local pollinator infectious disease dynamics than may have been previously thought.

Other industrialized agricultural livestock systems reflect extreme host densities similar to those in this study. However, the R_0_ for honeybee diseases may exceed that of other livestock diseases. We compare our lower threshold estimate for the R_0_ of *N. ceranae* to all available R_0_ values for livestock diseases that we could readily find in the literature (Figure S9, see S.I. Section 4). Notably, all other livestock diseases for which R_0_ estimates exist show minimum R_0_ values far below our honeybee estimate, however, examples of agricultural R_0_ values as high or higher than those we present for honeybees do also exist. There is, therefore, a clear need to develop explicit models of agricultural intensification scenarios for important agricultural disease.

Overall, our findings represent the first stage in developing robust epidemiological models for studying honeybee pathogens at an apiary scale. In the face of increasing challenges to global apiculture, our models predict that the size of apiaries per se is not causing notable increases in disease prevalence for important established bee pathogens, while it may increase the risk of pathogen emergence. Finally, this study demonstrates that conventional thought on how agricultural intensification influences disease may not be robust in the face of system‐specific ecological nuance.

## AUTHORS’ CONTRIBUTIONS

All authors contributed to conceptualization and scope definition of the study. L.J.B., C.R., M.B. developed approach. Mathematical modelling was undertaken by C.R., A.W. and M.B. Computational modelling by L.J.B., K.D., and M.B. Model scope and parameterization by L.J.B., K.D., J.C.d.R., B.J.B., L.W. L.J.B. and C.R. created figures, interpreted results and drafted manuscript with guidance and input from all authors. All authors contributed to further drafting, revision, and finalization. All authors approved the final version for publication.

## Data Availability

The agent‐based model is made available in association with this manuscript via Dryad Digital Repository https://doi.org/10.5061/dryad.rn2j5p0 (Bartlett et al., 2019).
