# Supplementary material for: Industrial bees: The impact of apicultural intensification on local disease prevalence
Source: J Appl Ecol. 2019 Jul 16;56(9):2195–205. doi: 10.1111/1365-2664.13461 (PMC6771535; doi:10.1111/1365-2664.13461)
Supplement: Supplementary file 1 [file JPE-56-2195-s001.pdf]

Supplementary Information for

Industrial bees: the impact of apicultural intensification on local disease prevalence".

Lewis J. Bartlett*, Carly Rozins*, Berry J. Brosi, Keith S. Delaplane, Jacobus C. de Roode, Andrew White, Lena Wilfert, Michael Boots

* These authors contributed equally to this work.

Correspondence:
ljbartl@emory.edu; crozins@berkeley.edu

This Supplementary Information includes:
Supplementary Text Sections 1 to 4
Equations [S1] to [S8]
Figs. S1 to S9
Table S1
References for SI citations

Supplementary Information Section 1

Mathematical Model

In this appendix we reintroduce the mathematical model, solve for the basic reproduction numbers, R_0 and approximate expressions for the endemic equilibrium. Additionally we show that R_0 can be used to determine the global stability of the system. When $R_0 > 1$ there is global asymptotic convergence to the endemic equilibrium and when $R_0 < 1$ there is global asymptotic convergence to the disease free equilibrium.

The Model

The following $2n$ differential equations, [1], model the transmission of disease within and between n subpopulations.

$$\begin{aligned} \frac{dS_i}{dt} &= -\sum_{j=1}^{n} S_i I_j \beta_{ij} - mS_i + \phi \\ \frac{dI_i}{dt} &= \sum_{j=1}^{n} I_j S_i \beta_{ij} - I_i(m+v) \end{aligned} \qquad \text{[S1]}$$

All parameter values are assumed to be nonnegative. The matrix $\beta = [\beta_{ij}]$ will depend on the arrangement of colonies within the apiary (see Fig. S1). It can be easily verified that each β (for each colony arrangement) is irreducible through the construction of the associated directional graphs (see Fig.1 and (1)). The feasible region for [S1]:

$$\Gamma = \left\{ (S_1, I_1, \ldots, S_n, I_n) \in \mathbb{R}_+^{2n} \middle| S_i + I_i \leq \frac{\phi}{m} \right\}, i = 1, 2, \ldots, n$$

is positively invariance with respect to [S1]. Let $\mathring{\Gamma}$ denote the interior of Γ. If there is no disease-causing pathogen then there exists a disease free equilibrium (DFE) $P_0 = (S_1^0, 0, S_2^0, 0, \ldots, S_n^0, 0)$ where, $S_i^0 = S^0 = \phi/m$, for $i = 1, 2, \ldots, n$ regardless of configuration (lattice, array, circle) or

population size (n). Below are examples for a 9-hive apiary, also see Fig. S1.

$$\text{array}\quad\begin{bmatrix} a & b & 0 & 0 & 0 & 0 & 0 & 0 & 0 \\ b & a & b & 0 & 0 & 0 & 0 & 0 & 0 \\ 0 & b & a & b & 0 & 0 & 0 & 0 & 0 \\ 0 & 0 & b & a & b & 0 & 0 & 0 & 0 \\ 0 & 0 & 0 & b & a & b & 0 & 0 & 0 \\ 0 & 0 & 0 & 0 & b & a & b & 0 & 0 \\ 0 & 0 & 0 & 0 & 0 & b & a & b & 0 \\ 0 & 0 & 0 & 0 & 0 & 0 & b & a & b \\ 0 & 0 & 0 & 0 & 0 & 0 & 0 & b & a \end{bmatrix} \qquad \text{circular}\quad\begin{bmatrix} a & b & 0 & 0 & 0 & 0 & 0 & 0 & b \\ b & a & b & 0 & 0 & 0 & 0 & 0 & 0 \\ 0 & b & a & b & 0 & 0 & 0 & 0 & 0 \\ 0 & 0 & b & a & b & 0 & 0 & 0 & 0 \\ 0 & 0 & 0 & b & a & b & 0 & 0 & 0 \\ 0 & 0 & 0 & 0 & b & a & b & 0 & 0 \\ 0 & 0 & 0 & 0 & 0 & b & a & b & 0 \\ 0 & 0 & 0 & 0 & 0 & 0 & b & a & b \\ b & 0 & 0 & 0 & 0 & 0 & 0 & b & a \end{bmatrix}$$

$$\text{lattice}\quad\begin{bmatrix} a & b & 0 & b & 0 & 0 & 0 & 0 & 0 \\ b & a & b & 0 & b & 0 & 0 & 0 & 0 \\ 0 & b & a & 0 & 0 & b & 0 & 0 & 0 \\ b & 0 & 0 & a & b & 0 & b & 0 & 0 \\ 0 & b & 0 & b & a & b & 0 & b & 0 \\ 0 & 0 & b & 0 & b & a & 0 & 0 & b \\ 0 & 0 & 0 & b & 0 & 0 & a & b & 0 \\ 0 & 0 & 0 & 0 & b & 0 & b & a & b \\ 0 & 0 & 0 & 0 & 0 & b & 0 & b & a \end{bmatrix}$$

Invasion - R_0 and local stability of the DFE

We use the *next generation matrix* (NGM) method (2, 3) to solve for the basic reproduction number, R_0. Let

$$V = \text{diag}(m+v, m+v, \ldots, m+v) \quad \text{and} \quad F(S) = \Big(S_i\beta_{ij}\Big)_{n\times n}, \qquad \text{[S2]}$$

be the $n \times n$ matrices for disease transition and new infections. Let:

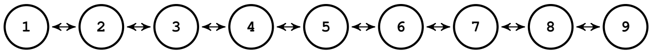

(a) array.

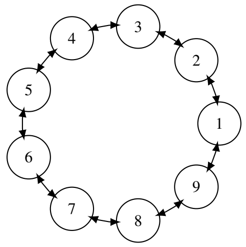

(b) circular.

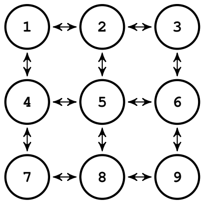

(c) lattice.

Figure S1. Schematics for sub-population configurations. Nodes represent sub-populations and edges represent possible modes for disease transmission

$$M(S) = F(S)V^{-1} = \left(\frac{\beta_{ij}S_i}{m+v}\right)_{n\times n} \quad \text{and} \quad M_0 = F(S^0)V^{-1} = \left(\frac{\beta_{ij}S^0}{m+v}\right)_{n\times n},$$

where M_0 is the NGM and the basic reproduction number, R_0, is defined as its spectral radius ($R_0 = \rho(M_0)$). Since β is irreducible, so too is $M(S)$ and M_0 as well as $M(S) + M_0$ and for $S^0 \neq S_i$, $M(S) < M_0$ and $\rho(M(S)) < \rho(M_0)$ (see (1)). Recall that the transmission matrix $\beta = [\beta_{ij}]$ differs for each population structure (array, circle, lattice), and thus so too will the spectral radius (R_0) of each unique NGM. Eigenvalues for the NGM are found in *Additional Information-Eigenvalues of the NGM* (at the end of this Supplementary Section).

$$R_0^{\text{Array}} = \frac{\phi}{m(m+v)}\left[a - 2b\cos(\frac{n\pi}{n+1})\right] \quad \text{[S3a]}$$

$$R_0^{\text{Circular}} = \frac{\phi}{m(m+v)}(a+2b) \quad \text{[S3b]}$$

$$R_0^{\text{Lattice}} = \frac{\phi}{m(m+v)}\left[a - 4b\cos(\frac{\sqrt{n}\pi}{\sqrt{n}+1})\right] \quad \text{[S3c]}$$

Global Dynamics of the DFE

In this section the global asymptotic stability of the DFE is established by constructing a suitable Lyapunov function. We use the matrix-theoretic approach, which is based on the Perron eigenvector of the NGM (see (1, 4, 5)).

Theorem 1. *The following holds for system [S1]:*

1. *If* $R_0 \leq 1$ *then the disease-free equilibrium is globally asymptotically stable in* Γ.

2. *If* $R_0 > 1$ *then the disease-free equilibrium is unstable and there exists an endemic equilibrium (EE),* $P^* = \{S_1^*, I_1^*, S_2^*, I_2^*, \ldots, S_n^*, I_n^*\}$

Proof. We proceed as in Proposition 3.1 in (1) and Theorem 4.1 in (5). Let $x = (I_1, I_2, \ldots I_n)^T$ and $F(S^0)$ and V are defined as in [S2]. Since $S^0 > S_i$, we have $x' < (F(S^0) - V)x$ and from Theorem 2.1 in (4) we have $L = w^T V^{-1} x$ is a global Lyapunov function for [S1], where w^T is

the left Perron eigenvector of the matrix $V^{-1}F(S^0)$ (the NGM).

$$
\begin{aligned}
L' &= w^T V^{-1} x' \\
&\leq w^T V^{-1}(F(S^0) - V)x \\
&= (w^T V^{-1} F(S^0) - w^T V^{-1} V)x \\
&= (R_0 - 1) w^T x \\
&\leq 0 \quad \text{if} \quad R_0 \leq 1
\end{aligned}
\qquad \text{[S4]}
$$

The only compact invariant subset, with respect to [S1], where $L' = 0$, is the singleton $\{P_0\}$. By LaSalle's Invariance Principle (6) P_0 is globally asymptotically stable in Γ if $R_0 \leq 1$. If $R_0 > 1$ and $x > 0$, then $(R_0 - 1)w^T x > 0$ and

$$L' = w^T V^{-1} x' = w^T V^{-1}(F(S) - V)x = w^T(M(S)x - x) > 0$$

in a neighbourhood of P_0 in $\mathring{\Gamma}$ by continuity. Thus P_0 is unstable. It can be shown that when $R_0 > 1$, the instability of P_0 implies the uniform persistence of [S1] (7), thus concluding the proof (see (1) for example). The existence of P^* follows from the uniform persistence and the positive invariance of the compact set (see Theorem 2.2 in (4) and Theorem 4.1 in (5)). □

Existence and Global Dynamics of the EE

In this section we show, with the use of a Lyapunov function, that when $R_0 > 1$ the EE, $P^* = \{S_1^*, I_1^*, S_2^*, I_2^*, \ldots, S_n^*, I_n^*\}$, is globally asymptotically stable. We will proceed as others have (1) using the graph theoretic approach.

Theorem 2. *If $\beta = [\beta_{ij}]$ is irreducible and $R_0 > 1$ then there exists a unique endemic equilibrium P^* that is globally asymptotically stable in $\mathring{\Gamma}$*

Proof. Set $\bar{\beta_{ij}} = \beta_{ij} S_i^* I_j^*$, for $1 \leq i, j, \leq n$, and $n \geq 2$ and let:

$$\overline{B} = \begin{bmatrix} \sum_{l\neq 1} \bar{\beta_{1l}} & -\bar{\beta_{21}} & \dots & -\bar{\beta_{n1}} \\ \bar{\beta_{12}} & \sum_{l\neq 2} \bar{\beta_{2l}} & \dots & -\bar{\beta_{n2}} \\ \vdots & \vdots & \ddots & \vdots \\ -\bar{\beta_{1n}} & -\bar{\beta_{2n}} & \dots & \sum_{l\neq 2} \bar{\beta_{nl}} \end{bmatrix}.$$

Where $\overline{B}$ is the transpose of the Laplacian matrix of the directional graph $\bar{\beta}$. Then by Lemma 2.1 in (1), a basis for the solution space for $\overline{B}u = 0$, where $u = (u_1, u_2, \dots, u_n)$, can be written as

$$(u_1, u_2, \dots, u_n) = (C_{11}, C_{22}, \dots, C_{nn}), \qquad \text{[S5]}$$

and

$$C_{ii} = \sum_{T \in \mathbb{T}_i} \prod_{(i,j)\in E(T)} \bar{\beta_{ij}},$$

where $\mathbb{T}_i$ is the set of all directed trees rooted at vertex i and $E(T)$ is the set of edge weights of the directed tree, T. By Kirchhoffs theorem C_{ii} is also the cofactor of the i-th diagonal entry of $\overline{B}$.

Set

$$L = \sum_{i=1}^{n} u_i (S_i - S_i^* \ln S_i + I_k - I_k^* \ln I_k). \qquad \text{[S6]}$$

Differentiating L and making use of right hand side of [S1] and the equilibrium conditions:

$$\phi = m S_i^* + \sum_{j=1}^{n} S_i^* I_j^* \beta_{ij} \;\text{ and }\; (m+v) I_i^* = \sum_{j=1}^{n} S_i^* I_j^* \beta_{ij}$$

we obtain,

$$
\begin{aligned}
L' &= \sum_{i=1}^{n} u_i\Big(S_i' - \frac{S_i^*}{S_i}S_i' + I_i' - \frac{I_i^*}{I_i}I_i'\Big)\\
&= \sum_{i=1}^{n} u_i\Big[\phi - mS_i - \sum_{j=1}^{n} S_iI_j\beta_{ij} - \frac{\phi S_i^*}{S_i} + S_i^*m + \sum_{j=1}^{n} S_i^*I_j\beta_{ij}\\
&+ \Big(\sum_{j=1}^{n} S_iI_j\beta_{ij}\Big) - (m+v)I_i + (m+v)I_i^* - \sum_{j=1}^{n}\frac{S_iI_jI_i^*\beta_{ij}}{I_i}\Big]\\
&= \sum_{i=1}^{n} u_i\Big[-S_i^*m\Big(\frac{S_i^*}{S_i} + \frac{S_i}{S_i^*} - 2\Big) + \Big(\sum_{j=1}^{n} S_i^*I_j\beta_{ij} - (m+v)I_i\Big)\\
&+ \Big(2\sum_{j=1}^{n} S_i^*I_j^*\beta_{ij} - \sum_{j=1}^{n}\frac{(S_i^*)^2I_j^*\beta_{ij}}{S_i} - \sum_{j=1}^{n}\frac{S_iI_jI_i^*\beta_{ij}}{I_i}\Big)\Big]
\end{aligned}
$$

Note that $\Big(\frac{S_i^*}{S_i} + \frac{S_i}{S_i^*} - 2\Big) \geq 0$, thus $-S_i^*m\Big(\frac{S_i^*}{S_i} + \frac{S_i}{S_i^*} - 2\Big) \leq 0$. Also

$$\sum_{i=1}^{n} u_i\Big(\sum_{j=1}^{n} S_i^*I_j\beta_{ij} - (m+v)I_i\Big) = 0$$

since $\sum_{i=1}^{n} u_i \sum_{j=1}^{n} \beta_{ij}S_i^*I_j = \sum_{j=1}^{n} u_j \sum_{i=1}^{n} \beta_{ji}S_j^*I_i = \sum_{i=1}^{n}\Big(\sum_{j=1}^{n} \beta_{ji}S_j^*u_j\Big)I_i$ and we can show that $\sum_{j=1}^{n} \beta_{ji}S_j^*u_j = u_i(m+v)$ using the equilibrium condition $(m+v)I_i^* = \sum_{j=1}^{n} S_i^*I_j^*\beta_{ij}$ and the following equality:

$$\begin{bmatrix} S_1^* I_1^* \beta_{11} + S_1^* I_2^* \beta_{12} + \cdots + S_1^* I_n^* \beta_{1n} \\ S_2^* I_1^* \beta_{21} + S_2^* I_2^* \beta_{22} + \cdots + S_2^* I_n^* \beta_{2n} \\ \vdots \\ S_n^* I_1^* \beta_{n1} + S_n^* I_2^* \beta_{n2} + \cdots + S_n^* I_n^* \beta_{nn} \end{bmatrix} = \begin{bmatrix} (m+v) I_1^* \\ (m+v) I_2^* \\ \vdots \\ (m+v) I_n^* \end{bmatrix}$$

$$\begin{bmatrix} S_1^* \beta_{11} & S_1^* \beta_{12} & \ldots & S_1^* \beta_{1n} \\ S_2^* \beta_{21} & S_2^* \beta_{22} & \ldots & S_2^* \beta_{2n} \\ \vdots & \vdots & & \vdots \\ S_n^* \beta_{n1} & S_n^* \beta_{n2} & \ldots & S_n^* \beta_{nn} \end{bmatrix} \begin{bmatrix} I_1^* \\ I_2^* \\ \vdots \\ I_n^* \end{bmatrix} = \begin{bmatrix} (m+v) & 0 & \ldots & \\ 0 & (m+v) & 0 & \ldots \\ \vdots & & \ddots & \\ & & & (m+v) \end{bmatrix} \begin{bmatrix} I_1^* \\ I_2^* \\ \vdots \\ I_n^* \end{bmatrix}$$

If we left multiply by u^T then we can show:

$$\begin{bmatrix} S_1^* u_1^* \beta_{11} + S_2^* u_2^* \beta_{21} + \cdots + S_n^* u_n^* \beta_{n1} \\ S_1^* u_1^* \beta_{12} + S_2^* u_2^* \beta_{22} + \cdots + S_n^* u_n^* \beta_{n2} \\ \vdots \\ S_1^* u_1^* \beta_{1n} + S_2^* u_2^* \beta_{2n} + \cdots + S_n^* u_n^* \beta_{nn} \end{bmatrix} = \begin{bmatrix} u_1(m+v) \\ u_2(m+v) \\ \vdots \\ u_n(m+v) \end{bmatrix}$$

and thus,

$$
\begin{aligned}
L' &\leq \sum_{i=1}^{n} u_i \Big(2 \sum_{j=1}^{n} S_i^* I_j^* \beta_{ij} - \sum_{j=1}^{n} \frac{(S_i^*)^2 I_j^* \beta_{ij}}{S_i} - \sum_{j=1}^{n} \frac{S_i I_j I_i^* \beta_{ij}}{I_i} \Big) \\
&= \sum_{i=1}^{n} u_i \Big(\sum_{j=1}^{n} 2\bar{\beta_{ij}} - \frac{s_i^* \hat{\beta_{ij}}}{S_i} - \frac{S_i I_j I_i^* \bar{\beta_{ij}}}{I_i S_i^* I_j^*} \Big) \\
&= \sum_{i=1}^{n} \sum_{j=1}^{n} u_i \bar{\beta_{ij}} \Big(2 - \frac{S_i^*}{S_i} - \frac{S_i I_j I_i^*}{I_i S_i^*} I_j^* \Big)
\end{aligned}
$$

It remains to show that $H_n \leq 0$ for all $(S_1, I_1, \ldots, S_n, I_n) \in \mathring{\Gamma}$. While explicit expressions for cofactors, u_i can be derived by computing the number of matrix trees for the diagonal entries in $\bar{\beta}$, it is difficult to do so for the lattice structure. Therefore we will direct the reader to (1, 8) and give a sketch of the remainder of their proof for a general irreducible transmission matrix β and associated matrix $\bar{\beta_{ij}} = \beta_{ij} S_i^* I_j^*$. Guo et al. (2006, 2008) show that $u_i = C_{ii}$ is the sum of n^{n-2} terms, each of which can be expressed as the product of $(n-1)$, $\bar{\beta_{ij}}$'s. Importantly the subindices of $\bar{\beta_{ij}}$ can be represented by all arcs in a directed tree T rooted at the $i-$th vertex. The product $u_i \bar{\beta_{ij}}$ can be interpreted as the weight of the unicycle graph, Q, obtained from the tree T, by adding an edge from node i to j. Each unicycle graph Q has a unique cycle CQ of length $1 \leq l \leq n$. Guo et al. show that there are l terms in H_n each with coefficients correspond to all $l-$rotations of the same $l-$cycle and are thus the same, and can be grouped together. Furthermore, all of the terms of H_n can be grouped based on corresponding cycle

lengths. Thus $H_n = \sum_Q H_{n,Q}$, where,

$$
\begin{aligned}
H_{n,Q} &= \prod_{(r,m)\in E(Q)} \bar{\beta}_{rm} \sum_{(i,j)\in E(CQ)} \big(2 - \frac{S_i^*}{S_i} - \frac{S_i I_j I_i^*}{I_i S_i^* I_j^*}\big) \\
&= \prod_{(r,m)\in E(Q)} \bar{\beta}_{rm} \big(2l - \sum_{(i,j)\in E(CQ)} \big(\frac{S_i^*}{S_i} - \frac{S_i I_j I_i^*}{I_i S_i^* I_j^*}\big)\big)
\end{aligned}
$$

where $E(CQ)$ is the edge weight of the cycle CQ and l denotes the number of edges in CQ. Note that

$$
\prod_{(i,j)\in E(CQ)} \big(\frac{S_i^*}{S_i} \frac{S_i I_j I_i^*}{I_i S_i^* I_j^*}\big) = \prod_{(i,j)\in E(CQ)} \frac{I_j I_i^*}{I_j^* I_i} = 1
$$

for each unicycle in Q. Therefore

$$
\sum_{(i,j)\in E(CQ)} \big(\frac{S_i^*}{S_i} - \frac{S_i I_j I_i^*}{I_i S_i^* I_j^*}\big) \geq 2l
$$

and thus $H_{n,Q} \leq 0$ for each Q and $H_{n,Q} = 0$ when $\frac{S_i^*}{S_j} = \frac{S_i I_j I_i^*}{I_i S_i^* I_j^*}$. Thus $H_, \leq 0$ and $L' \leq 0$ for all $(S_1, I_1, \ldots, S_n, I_n) \in \mathring{\Gamma}$ and $L' = 0$ iff $S_i = S_i^*$ and $H_n = 0$. Guo et al. (2006,2008) show that $H_n = 0 \Leftrightarrow I_i = aI_j^*$ where a is some arbitrary positive number. If we substitute $S_i = S^*$ and $I_j = aI_j^*$ into (S1), then

$$
0 = \phi - mS_i^* - a\sum_{j=1}^{n} S_i^* I_j^* \beta_{ij},
$$

holds true if $a = 1$ (i.e. at P^*), and otherwise the right hand side is strictly decreasing in a. Therefore the only compact invariant subset of the set where $L' = 0$ is the singleton $\{P^*\}$ and therefore by LaSalle Invariance Principle, P^* is globally stable in $\mathring{\Gamma}$ when $R_0 > 1$.

□

The Endemic Equilibrium

Equilibrium values for model [S1] are found by setting $dS_i/dt = dI_i/dt = 0$.

$$0 = -\sum_{j=1}^{n} S_i I_j \beta_{ij} - mS_i + \phi$$
$$0 = \sum_{j=1}^{n} I_j S_i \beta_{ij} - I_i(m+v)$$

For the circular configured model we can solve for the endemic equilibrium explicitly by solving the above for an apiary comprised of three colonies. This is done without loss of generality. The endemic equilibrium for the circular hive is:

$$(S^*, I^*) = \Big(\frac{m+v}{a+2b}, \frac{\phi}{m+v} - \frac{m}{a+2b}\Big)$$

If we assume that $0 < b << 1$, then we can express the solutions of the system of $2n$ nonlinear equations (above) each as a power series in b. This technique is refereed to as *perturbation theory*. For example, the power series solution for S_1 would be: $S_1(t) \approx S_1^0(t) + bS_1^1(t) + b^2 S_1^2(t) + b^3 S_1^3(t) + \dots$. We proceed by substituting the first two terms of each power series (i.e. $S_1 = S_1^0 + bS_1^1$, $S_2 = S_2^0 + bS_2^1$,...,$I_1 = I_1^0 + bI_1^1$,$I_2 = I_2^0 + bI_2^1$,... etc) into the the system of equations. We then collect equal powers of b, while neglecting higher powers (greater than 2). This results in two systems of of equations (one system for the variables with 0 as subscripts, and one system for the variables with both 0 and 1 as subscripts). The first system of equations (with variable superscripts 0, i.e. S_1^0, S_2^0,...,I_1^0, I_2^0,...) is easily solvable.

$$S_1^0 = S_2^0 = S_3^0 = ... = \frac{m+v}{a}$$

and

$$I_1^0 = I_2^0 = I_3^0 = ... = \frac{\phi}{m+v} - \frac{m}{a}$$

By substituting the solution to the fist set of equations into the second system of equations, the second system of equations can be expressed as a nonhomogeneous linear system of equations $b = Ax$, where:

$$A = \begin{bmatrix} P & Q \\ R & 0 \end{bmatrix}, \qquad b = \begin{bmatrix} l_1 S^0 I^0 \\ l_2 S^0 I^0 \\ l_3 S^0 I^0 \\ \vdots \\ -l_1 S^0 I^0 \\ -l_2 S^0 I^0 \\ -l_3 S^0 I^0 \\ \vdots \end{bmatrix}, \qquad x = \begin{bmatrix} S_1^1 \\ S_2^1 \\ S_3^1 \\ \vdots \\ I_1^1 \\ I_2^1 \\ I_3^1 \\ \vdots \end{bmatrix}$$

The vector b is what differs between the array and the lattice models. The coefficients l_i are the number of neighbors that hive i has. For example, in the array configuration, hive one will have one neighbor, thus $l_1 = 1$ and hive two will have two neighbors $l_2 = 2$. The matrices P, Q, R and S are all $n \times n$ diagonal matrices, with zeros in all entries but the main diagonal. $P = \text{diag}(-aI^0 - m) = \frac{-a\phi}{m+v}$, $Q = \text{diag}(-aS^0) = -(m+v)$ and $R = \text{diag}(aI^0) = \frac{a\phi - m(m+v)}{m+v}$. The solution to the non-homogeneous linear system can be found by inverting the matrix A: $A^{-1}b = x$. The inverse of A is can be expressed in block form.

$$A^{-1} = \begin{bmatrix} 0 & C^{-1} \\ B^{-1} & AB^{-1}C^{-1} \end{bmatrix}$$

and

$$x = \begin{bmatrix} S_1^1 \\ S_2^1 \\ S_3^1 \\ \vdots \\ I_1^1 \\ I_2^1 \\ I_3^1 \\ \vdots \end{bmatrix} = \begin{bmatrix} \frac{-l_1(m+v)}{a^2} \\ \frac{-l_2(m+v)}{a^2} \\ \frac{-l_3(m+v)}{a^2} \\ \vdots \\ \frac{l_1 m}{a^2} \\ \frac{l_2 m}{a^2} \\ \frac{l_3 m}{a^2} \\ \vdots \end{bmatrix}$$

Therefore, the linear approximate endemic equilibrium is:

$$\begin{aligned} S_i^* &= \frac{m+v}{a} - lb\Big(\frac{m+v}{a^2}\Big) \\ I_i^* &= \frac{\phi}{m+v} + lb\Big(\frac{m}{a^2}\Big) \end{aligned} \qquad [S7]$$

where l is the number of neighbours that hive i has. Notice that the endemic equilibrium for a single colony is independent of the total number of colonies in the apiary. However, as the number of colonies increases, the average number of nearest neighbours any given colony has approaches a constant (2 for array and 4 for lattice). Thus, as the number of colonies increase, the populations-wide disease prevalence asymptotes.

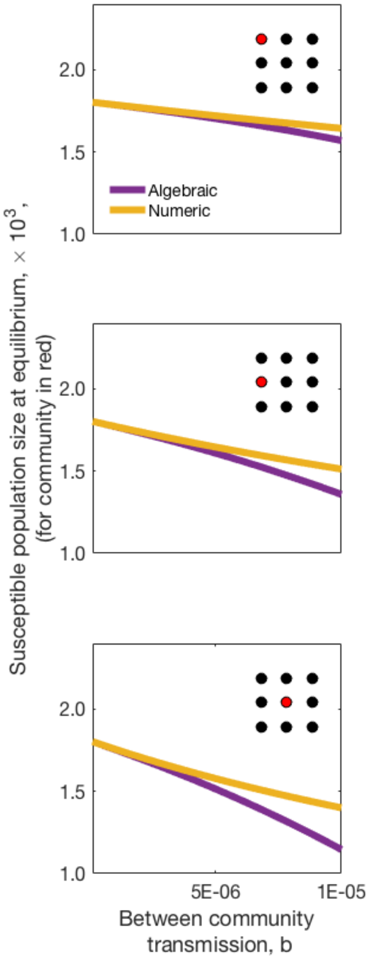

Figure S2. Analytic and numeric approximations for the susceptible endemic equilibrium population for three subpopulations and a range of between population transmission values, b. Other parameters are set to: $v = 0.16$, $m = 0.0275$, $\phi = 1600$ and finally the totoal transmission $T = a + b$ is held at $T = 1.04 \times 10^{-4}$

Robustness of EE approximations

The endemic equilibrium solutions found in the previous section rely on the between colony transmission being small. Therefore as the between colony transmission increases, the result [S7] will become less accurate (see Fig S2). Additionally, as the number of nearest neighbours

increases, so too does the discrepancy between the analytic and numeric results (Fig. S2). In Figure S2 we plot the endemic susceptible population size for particular colony within a nine-community lattice population. In yellow is the numeric solution and in purple is the algebraic solutions [S7]. You can see that as the parameter b, between colony transmission, increases, the two solutions diverge and the algebraic solution becomes an underestimate of the true susceptible populations size. Total transmission $a + b$ is held constant - when we increase b we also decreased a. As the between colony transmission proportion of the total transmission increases, the endemic susceptible populations size decreases (Fig S2) and the disease prevalence increases.

Additional Information- Eigenvalues of the NGM

Array Configuration

The next generation matrix for the array configuration model is:

$$FV^{-1} = \frac{\phi}{m(m+v)} \begin{bmatrix} a & b & & \\ b & a & \ddots & \\ & \ddots & \ddots & \\ & & & \end{bmatrix}$$

The matrix FV^{-1} is a tridiagonal Toeplitz matrix. Toeplitz matrices have been widely studied (9) and the eigenvalues of FV^{-1} are:

$$\lambda_k = \frac{\phi}{m(m+v)} \left[a - 2b\cos(\frac{k\pi}{n+1}) \right] \qquad \text{[S8]}$$

where $k = 1, .., n$ (9).

Lemma 1. *The largest values of the sequence,*

$$f_k = a - 2bcos\Big(\frac{k\pi}{n+1}\Big)$$

where $k = 1, 2, \ldots, n$ *and* $0 < b << a$ *is when* $k = n$.

Proof. We will prove through contradiction. Suppose that the maximum element of f_k is not f_n (the last element in the sequence). Then there must exist a $m \in 1, 2, ...(n-1)$ such that $f_m > f_n$.

$$a - 2b\cos\left(\frac{m\pi}{n+1}\right) > a - 2b\cos\left(\frac{n\pi}{n+1}\right)$$
$$\cos\left(\frac{m\pi}{n+1}\right) < \cos\left(\frac{n\pi}{n+1}\right)$$
$$\left(\frac{m\pi}{n+1}\right) > \left(\frac{n\pi}{n+1}\right)$$
$$m > n$$

which is a contradiction. Hence the largest element of f_k is f_n.

□

Therefore the dominant eigenvalue of FV^{-1}, and thus R_0 for the array model is:

$$R0^{\text{array}} = \frac{\phi}{m(m+v)}\Big[a - 2b\cos(\frac{n\pi}{n+1})\Big]$$

Circular Configuration

We proceed as we did above. For the circular model,

$$FV^{-1} = \frac{\phi}{m(m+v)} \begin{bmatrix} a & b & 0 & \cdots & b \\ b & a & b & \cdots & \\ & \cdots & \cdots & & \\ & & & & \end{bmatrix}$$

Again FV^{-1} is a Toeplitz matrix, but it is not tridiagonal. Unlike the matrix for the array model, the above matrix is a special class of Toeplitz matrices called a circular matrix where each row vector is rotated one element to the right relative to the preceding row vector. We denote the elements of the first row as $c_0, c_1, \ldots, c_{n-1}$, and note that regardless of what n is, $c_0 = a(\phi/(m(m+v)))$, $c_1 = b(\phi/(m(m+v)))$ and $c_{n-1} = b(\phi/(m(m+v)))$ while $c_j = 0$ where $j = (l \in \mathbb{N} | l < (n-1), l \neq 0, 1)$. The n eigenvalues of our circular matrix, FV^{-1}, are:

$$\lambda_k = \sum_{j=0}^{n-1} c_j e^{\frac{-2\pi i k j}{n}}$$

where c_j of the kth element of the top row of the matrix FV^{-1}. Notice that the top row of FV^{-1} has only three nonzero elements $c_0 = a(\phi/(m(m+v)))$, $c_1 = b(\phi/(m(m+v)))$ and $c_{n-1} = b(\phi/(m(m+v)))$. Therefore we can rewrite the above:

$$\begin{aligned} \lambda_k &= \frac{\phi}{m(m+v)} \Big[a + be^{\frac{-2\pi \imath k}{n}} + be^{\frac{-2(n-1)\pi \imath k}{n}} \Big] \\ &= \frac{\phi}{m(m+v)} \Big[a + 2b \Big[\cos\Big(\frac{-2\pi k}{n} \Big) \Big] \Big] \end{aligned}$$

Note that $\sin\left(\frac{-2\pi k}{n}\right) + \sin\left(\frac{-2\pi k(n-1)}{n}\right) = 0$, since the function is periodic with period 2π and $\cos\left(\frac{-2\pi k}{n}\right) = \cos\left(\frac{-2\pi k(n-1)}{n}\right)$ since $\cos$ is periodic with period 2π and also a odd function.

We can proceed just as we did in the previous section to show that the dominant eigenvalue (and hence R_0) for the circular model is:

$$R0^{\text{Circular}} = \frac{\phi}{m(m+v)}(a+2b)$$

Lattice Configuration

To find R_0 for the lattice model we will make use of the Kronecker product and Kronecker sum. Consider the next generation matrix for the lattice model:

$$FV^{-1} = \frac{\phi}{m(m+v)} \begin{bmatrix} A & B & & \\ B & A & \ddots & \\ & \ddots & \ddots & \\ & & & \end{bmatrix}$$

$FV^{-1} \in \mathbb{M}^{n,n}$, and

$$A = \begin{bmatrix} a & b & & \\ b & a & \ddots & \\ & \ddots & \ddots & \\ & & & \end{bmatrix}, \qquad B = \begin{bmatrix} 0 & b & & \\ b & 0 & \ddots & \\ & \ddots & \ddots & \\ & & & \end{bmatrix}$$

where $A \in \mathbb{M}^{N,N}$ and $B \in \mathbb{M}^{N,N}$, $n = N^2$ and both A and B are tridiagonal Toeplitz matricies. Let

$$M = \begin{bmatrix} A & B & & \\ B & A & \ddots & \\ & \ddots & \ddots & \\ & & & \end{bmatrix}$$

The matrix M can be written as:

$$M = (B \otimes I) + (I \otimes A) = B \oplus A$$

Both A and B are Toeplitz with eigenvalues $-2b\cos(\frac{k\pi}{N+1})$ and $a - 2b\cos(\frac{k\pi}{N+1})$ for $k = 1, 2, ..., N$ respectively.

The n eigenvalues of M are:

$$\Big(-2b\cos(\frac{k\pi}{N+1})\Big) + \Big(a - 2b\cos(\frac{l\pi}{N+1})\Big)$$

for $k = 1, \dots N$ and $l = 1, \dots N$. The above is maximized when both $k = N = \sqrt{n}$ and $l = N = \sqrt{n}$ (See Lemma). Therefore R_0 for the lattice model is:

$$R0^{\text{Lattice}} = \frac{\phi}{m(m+v)}\Big[\Big(-2b\cos(\frac{\sqrt{n}\pi}{\sqrt{n}+1})\Big) + \Big(a - 2b\cos(\frac{\sqrt{n}\pi}{\sqrt{n}+1})\Big)\Big]$$

References - Section 1

1. H. Guo, M. Li, and Z. Shuai, “Global stability of the endemic equilibrium of multigroup sir epidemic models,” *Canadian Applied Mathematics Quarterly*, vol. 14, no. 3, pp. 259–284, 2006.

2. P. Van den Driessche and J. Watmough, “Reproduction numbers and sub-threshold endemic equilibria for compartmental models of disease transmission,” *Mathematical biosciences*, vol. 180, no. 1, pp. 29–48, 2002.

3. O. Diekmann, J. Heesterbeek, and J. Metz, “On the definition and the computation of the basic reproduction ratio r0 in models for infectious diseases in heterogeneous populations,” *Journal of mathematical biology*, vol. 28, no. 4, pp. 365–382, 1990.

4. Z. Shuai and P. van den Driessche, “Global stability of infectious disease models using lyapunov functions,” *SIAM Journal on Applied Mathematics*, vol. 73, no. 4, pp. 1513–1532, 2013.

5. Z. Shuai and P. van den Driessche, “Modelling and control of cholera on networks with a common water source,” *Journal of biological dynamics*, vol. 9, no. sup1, pp. 90–103, 2015.

6. J. LaSalle, *The stability of dynamical systems*, vol. 25. Siam, 1976.

7. H. Freedman, S. Ruan, and M. Tang, “Uniform persistence and flows near a closed positively invariant set,” *Journal of Dynamics and Differential Equations*, vol. 6, no. 4, pp. 583–600, 1994.

8. H. Guo, M. Li, and Z. Shuai, “A graph-theoretic approach to the method of global lyapunov functions,” *Proceedings of the American Mathematical Society*, vol. 136, no. 8, pp. 2793–2802, 2008.

9. D. Kulkarni, D. Schmidt, and S. Tsui, “Eigenvalues of tridiagonal pseudo-toeplitz matrices,” *Linear Algebra and its Applications*, vol. 297, no. 1-3, pp. 63–80, 1999.

Supplementary Information Section 2 – Agent Based Model

The ABM is made publicly available both on Dryad Digital Repository (see main manuscript) and accessible in GitHub under the following link: https://github.com/LBartlett/IndustrialBees2019

Description of our Agent-Based Model

We use a discrete time simulation with time-steps of 1 day. We define an apiary of size *n* where *n* is the number of colonies, arranged in one of three configurations following Fig. 1 in the main manuscript.

Within a colony, individuals are either susceptible (S) or infected (I). New susceptible individuals enter the colony at birth rate ϕ. Each colony has a constant birth rate ϕ, randomly drawn from a normal distribution with mean ϕ and $\sigma^2 = \phi \times 0.1$, typically $\phi = 1600$ in line with quoted maximum laying rates (1). We fix mean maximum colony size in a disease free state as M, typically 58200 individuals (2), and from this calculate a universal natural death rate $m = M/\phi$ (likelihood per individual per day). Death rates using our typical values are in line with rates quoted for various honeybee life stages (1, 3, 4). Differences in birth rate cause colonies to reach different maximum sizes in their disease free state, meant to approximate differences in queen quality (5), but is likely conservative in this regard.

Our starting state at time t = 0 is intended to represent the beginning of a beekeeping season. Each colony has a starting number of susceptible individuals, randomly drawn from a normal distribution with mean $S_{t=0} = 9 \times \phi$ and $\sigma^2 = 9/8 \times \phi$. Notably this would typically be well below the maximum colony size, as would be more realistic following overwintering (2). One colony in the apiary is randomly selected, and a single susceptible individual replaced with an infected individual.

Infected individuals inside a colony infect susceptible individuals at rate β. Infected individuals suffer an additional induced mortality rate *v*. We vary the values of β and *v* as part of this study. All individuals in a spatially structured population may additionally move into nearest-neighbouring colonies for a single time step at rate ρ – in the case of the lattice, we used a Von Neumann neighbourhood. We vary movement-rate ρ during this study, with minimum realistic rates derived from various other studies (4, 6, 7) and corrected for our lack of internal colony demography. Susceptible individuals which move into neighbouring colonies are not available to be infected within their own colony in that time step, but may become infected by infected individuals in the neighbouring colony to which they have temporarily moved. Likewise, infected individuals which have moved cannot contribute to infection within their own colony in this time step, but can infect susceptible individuals in the colony to which they have moved. The likelihood of movement into another colony ρ is per bee per day. Individuals which move between colonies remain residents of their 'home colony' and do not permanently become individuals in the colony to which they drifted for

the day. In the case of the fully mixed model, we relax the nearest neighbour assumption, and drift occurs randomly across the whole apiary.

Each time step, the above described processes of birth, death, and infection are modelled to occur simultaneously within and across all colonies in the apiary. Notably, this means that new susceptible individuals do not contribute to any other processes in the same time step in which they are born – they cannot die, move into another colony or become infected. This is the main driver of slightly lower prevalences observed in the stochastic simulations compared to the agent-based model. Additionally, individuals can die and contribute to infection in the same time step.

For this study, the agent-based model was built and run using R (v. 3.3.0 "Supposedly Educational") (8).

The agent-based model is qualitatively and conceptually the same as the analytical model and can be understood by using the following relationships between the two model parameterisations: $\beta \approx a + b$ and $\rho \approx b / (a + b)$.

References – Section 2

1. Schmickl T, Crailsheim K (2007) HoPoMo: A model of honeybee intracolonial population dynamics and resource management. *Ecol Model* 204(1):219–245.

2. Caron DM, Connor LJ (2013) *Honey Bee Biology and Beekeeping, Revised Edition* eds Muir RG, Harman A (Wicwas Press, Kalamazoo, MI). Revised edition.

3. Martin SJ (2001) The role of Varroa and viral pathogens in the collapse of honeybee colonies: a modelling approach. *J Appl Ecol* 38(5):1082–1093.

4. Becher MA, et al. (2014) BEEHAVE: a systems model of honeybee colony dynamics and foraging to explore multifactorial causes of colony failure. *J Appl Ecol* 51(2):470–482.

5. Hatjina F, et al. (2014) A review of methods used in some European countries for assessing the quality of honey bee queens through their physical characters and the performance of their colonies. *J Apic Res* 53(3):337–363.

6. Allen MD (1963) Drone production in honey-bee colonies (Apis mellifera L.). *Nature* 199:789–790.

7. Wilkinson D, Smith GC (2002) A model of the mite parasite, Varroa destructor, on honeybees (Apis mellifera) to investigate parameters important to mite population growth. *Ecol Model* 148(3):263–275.

8. R Core Team (2018) *R: A language and environment for statistical computing.* (R Foundation for Statistical Computing, Vienna, Austria) Available at: https://www.R-project.org/.

Supplementary Information Section 3

Additional Analysis

Model outputs were initially tested in mapping the effects of intensification to disease burden without consideration of R_0. This appendix shows select outputs confirming model function, supporting assertions made in the main text, and that the agent based models yield the same qualitative results as are derived from the mathematical model in the main text.

Figure S3 explores endemic equilibrium states for four pathogen phenotypes, for the which the R_0 values have been retrospectively approximated. Of note is the confirmation that higher R_0 yields higher pathogen burdens. By separately varying both virulence and transmission, we demonstrate the expected result that the highest prevalence is achieved by a low-virulence, high-transmission (i.e. 'well adapted') pathogen. The greatest degree of colony-size suppression results from a high-virulence, high-transmission pathogen, which is used as the pathogen in the subsequent figures.

Figure S4 shows how increasing movement between colonies (ρ) affects pathogen spread via mean colony sizes and pathogen prevalence for large or small apiaries in all configurations. As expected from the explanation and results presented in the main text, all aspects of intensification have little influence on the equilibrium disease burden (prevalence & colony size, which are positively related. Notably, for any given pathogen, it demonstrates that prevalence is directly relatable to colony size. We use this as justification for explicitly examining burden as the main focus of the manuscript. Additionally, it shows the very rapid rate at which the system reaches disease equilibrium for all apiaries in lattice configurations, and all small apiaries. The cases of spread in large circular or array apiaries are somewhat slower (and slightly influenced by higher rates of movement between colonies) but may be limited by our limitation of nearest-neighbour-only transmission. Again, we use this rapid rate of pathogen spread to disease equilibrium to justify our focus on disease endemic states in the main manuscript.

Figure S5 demonstrates the behaviour of the mathematical model in reaching disease equilibrium. Even in a large apiary of 100 colonies arranged in an array, endemic equilibrium is quickly established. This is in broad agreement with the results presented in Fig S4, with some minor differences potentially due to the agent based simulation being restricted to 1 day time steps (a constraint absent from the mathematical model). Our rate of spread present here (time taken to reach endemic equilibrium) may be a conservative estimate of reality, as we restrict inter-colony transmission to be between nearest neighbour colonies only.

Figure S6 shows a singular comparison of colony configurations in more detail, using the agent-based model for a single parameter set. We see rapid reaching of the equilibrium and qualitatively mirrored behaviour between the ABM and mathematical model. Additionally, fig. S6 shows that the relaxation of the 'nearest neighbour assumption' – looking at the extreme case where drifting is throughout the whole apiary – doesn't change model behaviour in a meaningful way.

Figure S7 Demonstrates that the model reaches the equilibrium rapidly. This is the case for the SI model presented in the main document, as well as for an alternative model where only larvae are vulnerable to infection by infectious adult bees.

Figure S8 uses purely the analytical mathematical model to examine the impact of aspects of intensification on burden in a similar approach to the results shown in main manuscript Fig. 5b. However it does not examine 'intensification' as one combined process and instead shows different combinations of numbers of colonies and configurations, with bee movement between colonies held constant. This figure (like Figure S5) does not involve the agent-based model results and should be understood as a test of consistency of results when comparing between modelling approaches.

Alternative Model

The alternative model has two age classes, Larvae (L) and Adults (A). Larvae develop into adults at rate g and die are rate m'. Larvae can become infected through contact with infected adults (carrying mites), A_I, with transmission rate of beta. Infected Adults have an additional death rate of v and all adults have a natural mortality of m; Infected adults can recover from infection at rate gamma. Demonstration of model behaviour is shown in Fig. S8.

$$\frac{dL_S}{dt} = \phi - m'L_S - gL_S - \beta A_I L_S$$

$$\frac{dL_I}{dt} = -m'L_I - gL_I + \beta A_I L_S$$

$$\frac{dA_S}{dt} = gL_S - mA_S + \gamma A_I$$

$$\frac{dA_I}{dt} = gL_I - (m + v)A_I - \gamma A_I$$

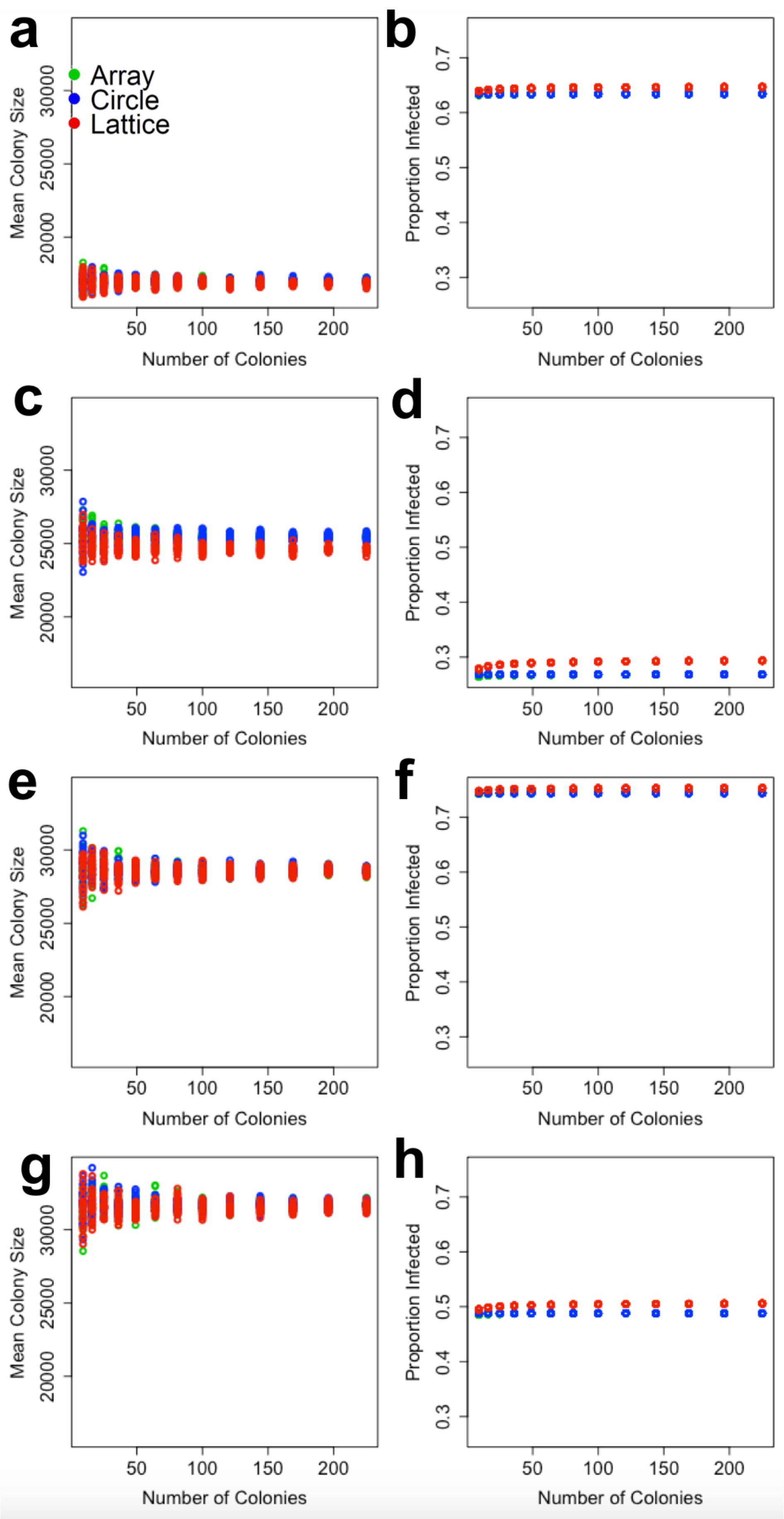

Figure S3. Graphs showing approximate endemic disease equilibria states expressed as mean colony size (left – a, c, e, g) and proportion of individuals infected (right – b, d, g, h) across a apiaries of varying sizes after 2000 days of simulation. Each row represents a different pathogen phenotype, and therefore R_0. Figs. a & b show equilibria for a high-mortality high-transmission pathogen ($R_0 \simeq 18$). Figs. c & d show a high-mortality low-transmission pathogen ($R_0 \simeq 2.5$); figs. e & f represent a low-mortality high-transmission pathogen (R0 $\simeq$ 36); and a low-mortality low-transmission pathogen ($R_0 \simeq 7.5$) is represented in figs. g & h. These prevalences can be compared to the relationship derived by the purely analytical model (Fig. 3c, main manuscript) between R_0 and prevalence, demonstrating the close agreement of the agent based model and its mathematical counterpart (see Fig. 4a).

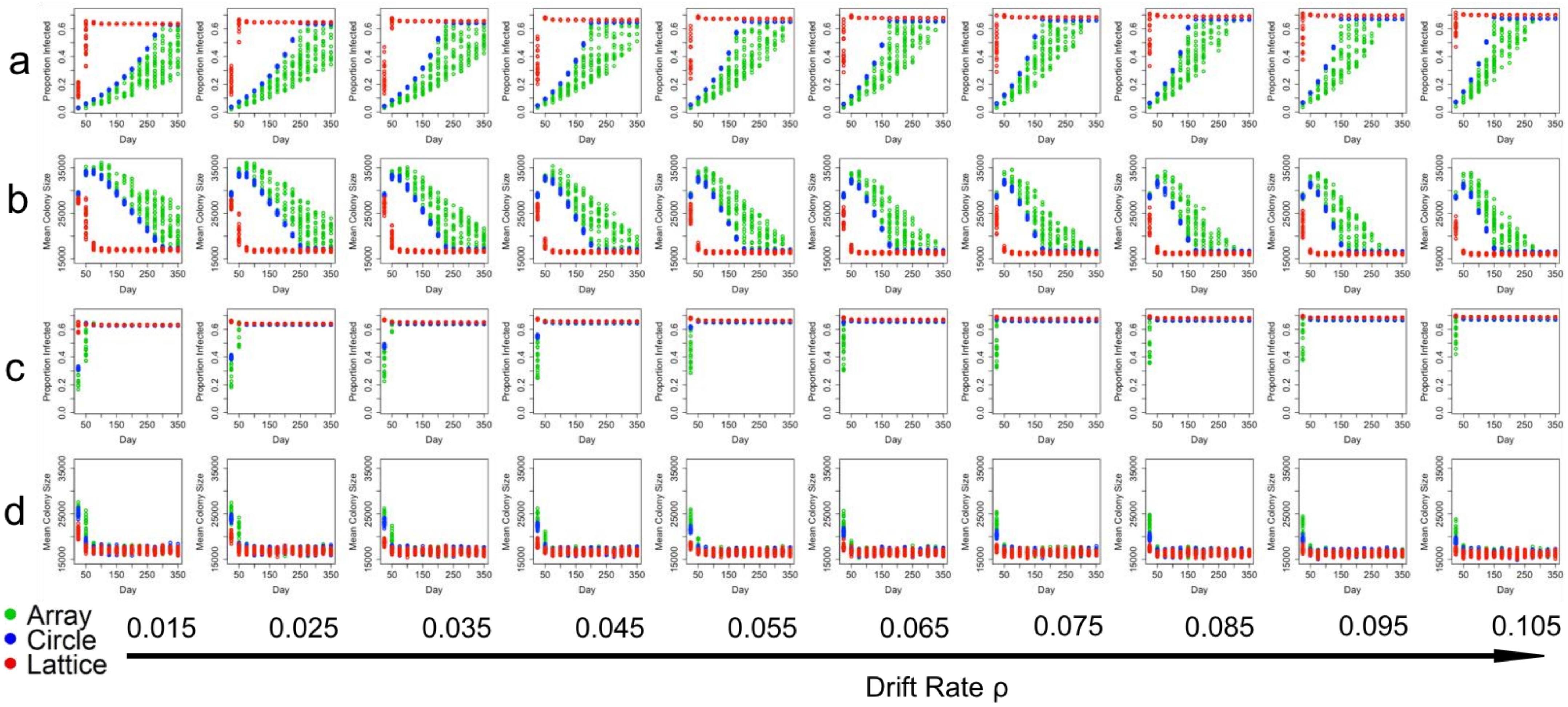

Figure S4. Graphs show change in mean colony size (rows b & d) and proportion of bees infected (rows a & c) across an apiary over the first 300 days of simulation, representing pathogen spread from a single colony. Rows a & b (top) show a large apiary (144 colonies), rows c & d (bottom) show a small apiary (16 colonies). Movement rate ρ increases from left to right. Corresponding R_0 values for these scenarios range from $R_0 \simeq 18$ at $\rho = 0.015$ in the small array apiary to $R_0 \simeq 30$ at $\rho = 0.0105$ in the large lattice apiary.

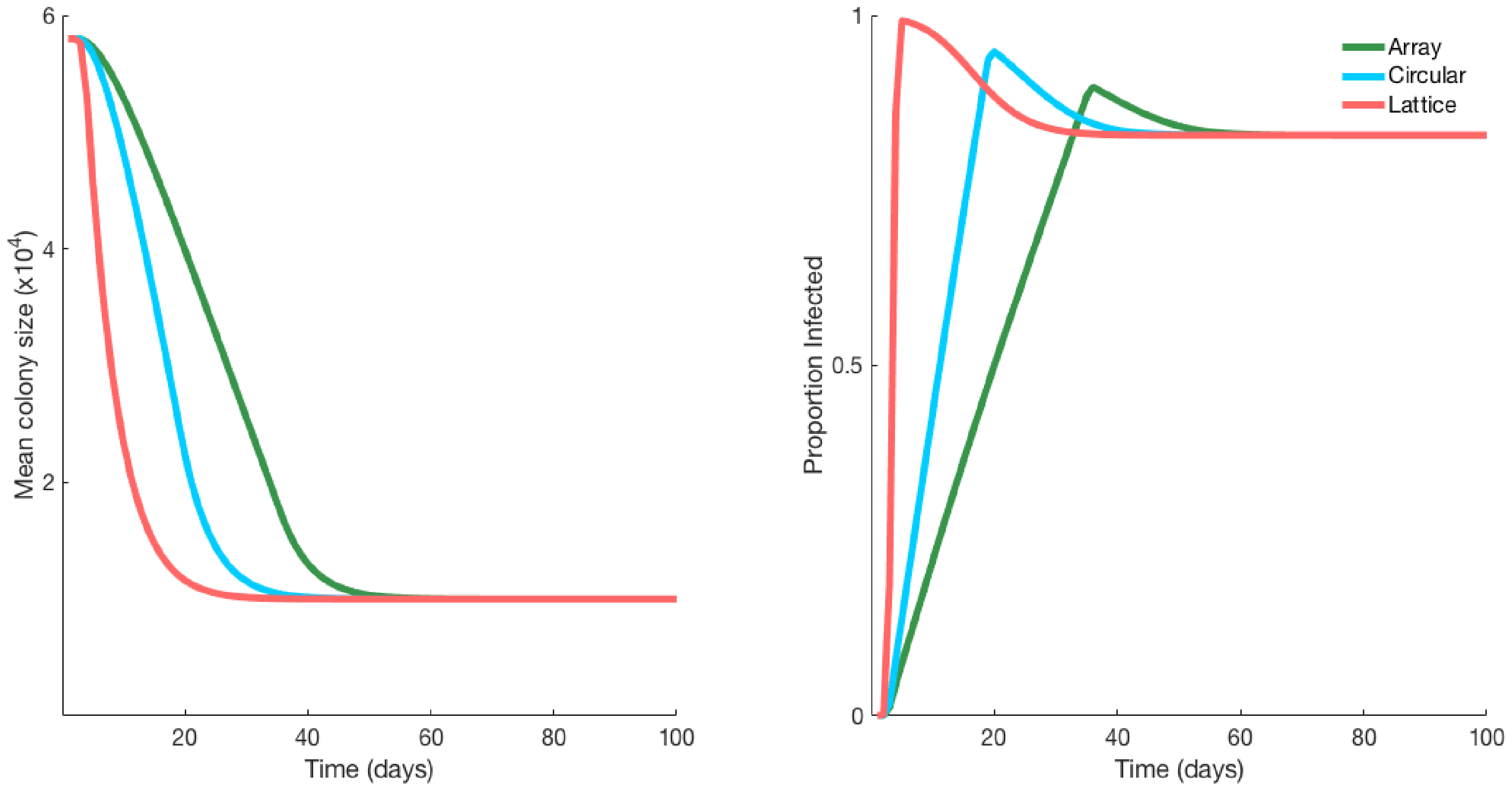

Figure S5. Graphs obtained from the mathematical model showing the rapid rate of spread throughout an apiary, where endemic disease equilibrium is quickly reached. Graphs show the case for each apiary arrangement (Array, Circular, Lattice) for an apiary of 100 colonies and movement rate between colonies of 0.02. The model starts with a single bee infected in one colony. The left panel shows the mean colony size, which closely matches the right panel showing proportion infected (compare also to the agent based simulation outputs Figs. S3 and S4.

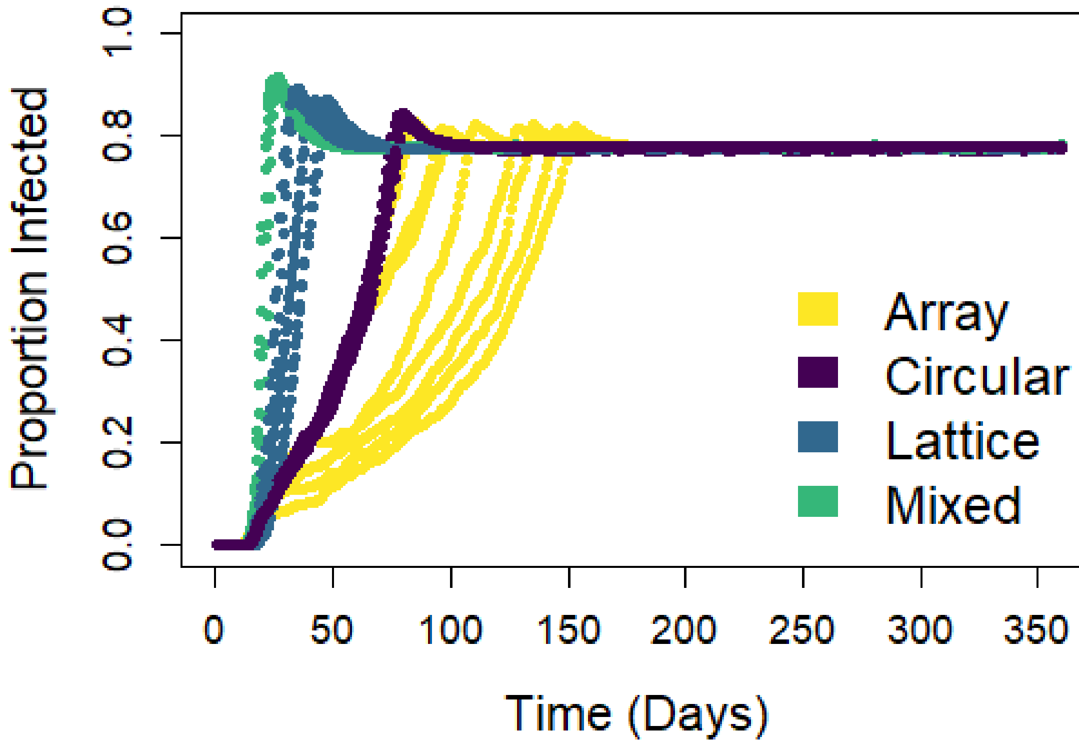

Figure S6. ABM data showing a single parameter set comparison of the three apiary configurations compared to a mixed model where the ‘nearest neighbor’ assumption is relaxed. Notably, this more detailed view of the dynamics shows strong agreement with Fig. S5 (above) taken from the analytical model. Additionally, we see that the ‘nearest neighbour’ assumption doesn’t meaningfully chance model performance – the magnitude of difference between the lattice and the mixed model notably smaller than between the lattice and the circular or array configurations. Each configuration was replicated eight times, under the following parameter set: n = 64, M = 58200, $\phi = 1600$, $\beta = 5x10^{-5}$, $\nu = 0.1$, $\rho = 0.05$.

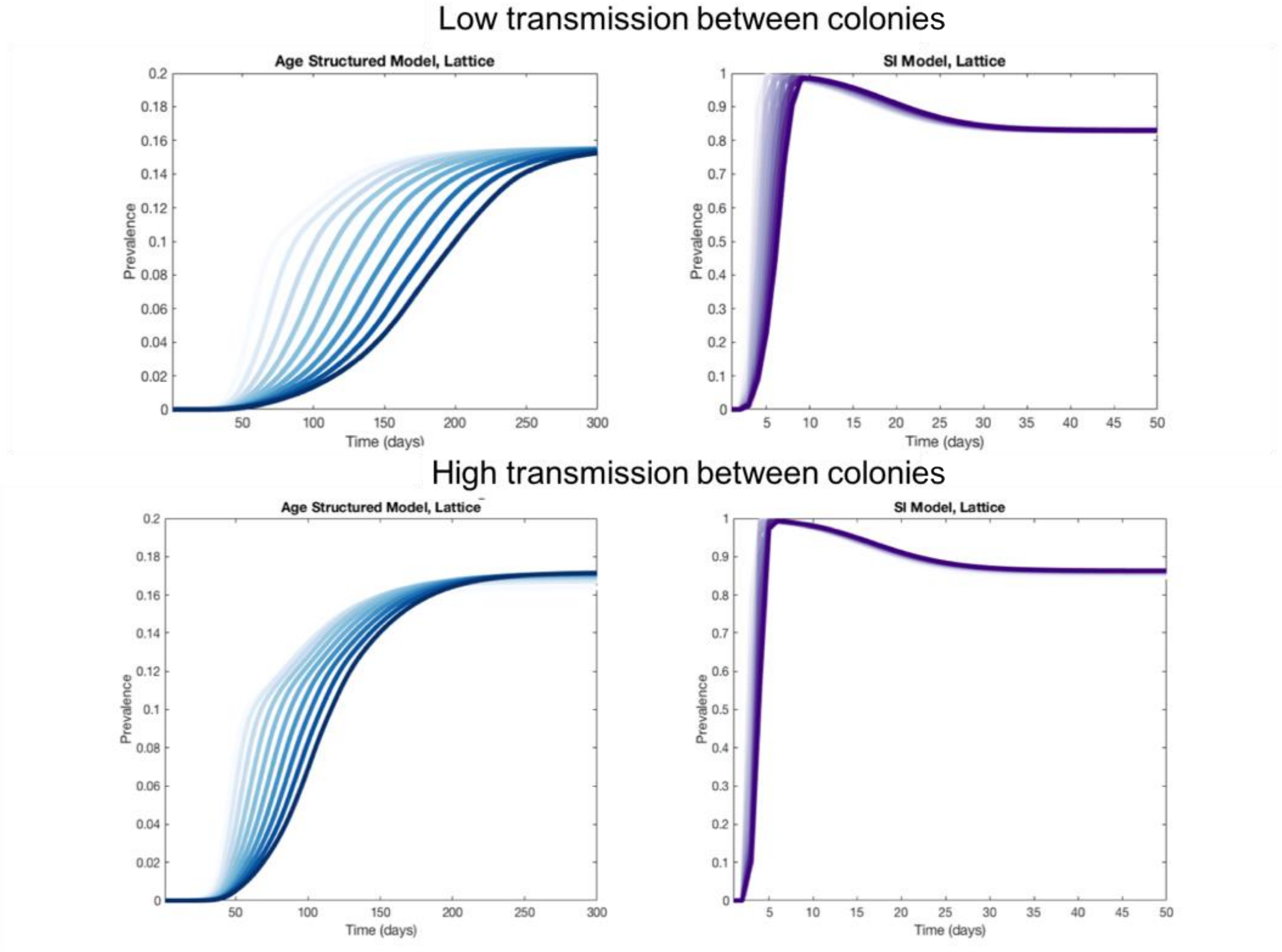

Figure S7. Comparison of the alternative (age-structured) model (left) behaviour and main (SI) model (right). Note both axes differ in scales. Plotted is the proportion of adult bees infected over time for the age structured model and proportion of all bees infected for the SI model, for ten apiary sizes (number of colonies) and two different inter-colony transmission rates (bee movement between colonies). The ten lines of different hues in each colour represent a unique number of colonies per apiary. Lightest colour represents an apiary with four hives and the darkest line represents an apiary with 121 hives. All simulations are for a lattice structure. The age-structured model shows much slower convergence than the SI model, however still converges in all cases in a single-season timescale. The largest apiaries in all cases take the longest amount of time to converge. Faster convergence is seen for the higher transmission rate between colonies, and slightly higher prevalences are apparent in the age-structured model for larger apiaries and higher inter-colony transmission rates.
Alternative model: phi=1600, a= a=1.04x10-4, b=0.1*a or b=0.018*a, v=0.16, m=0.033, m'=0.01,gamma=0.1,g=0.0476 .
SI model: phi=1600, a=1.04x10-4, b=0.1*a or b=0.018*a, v=0.16, m=0.0275.

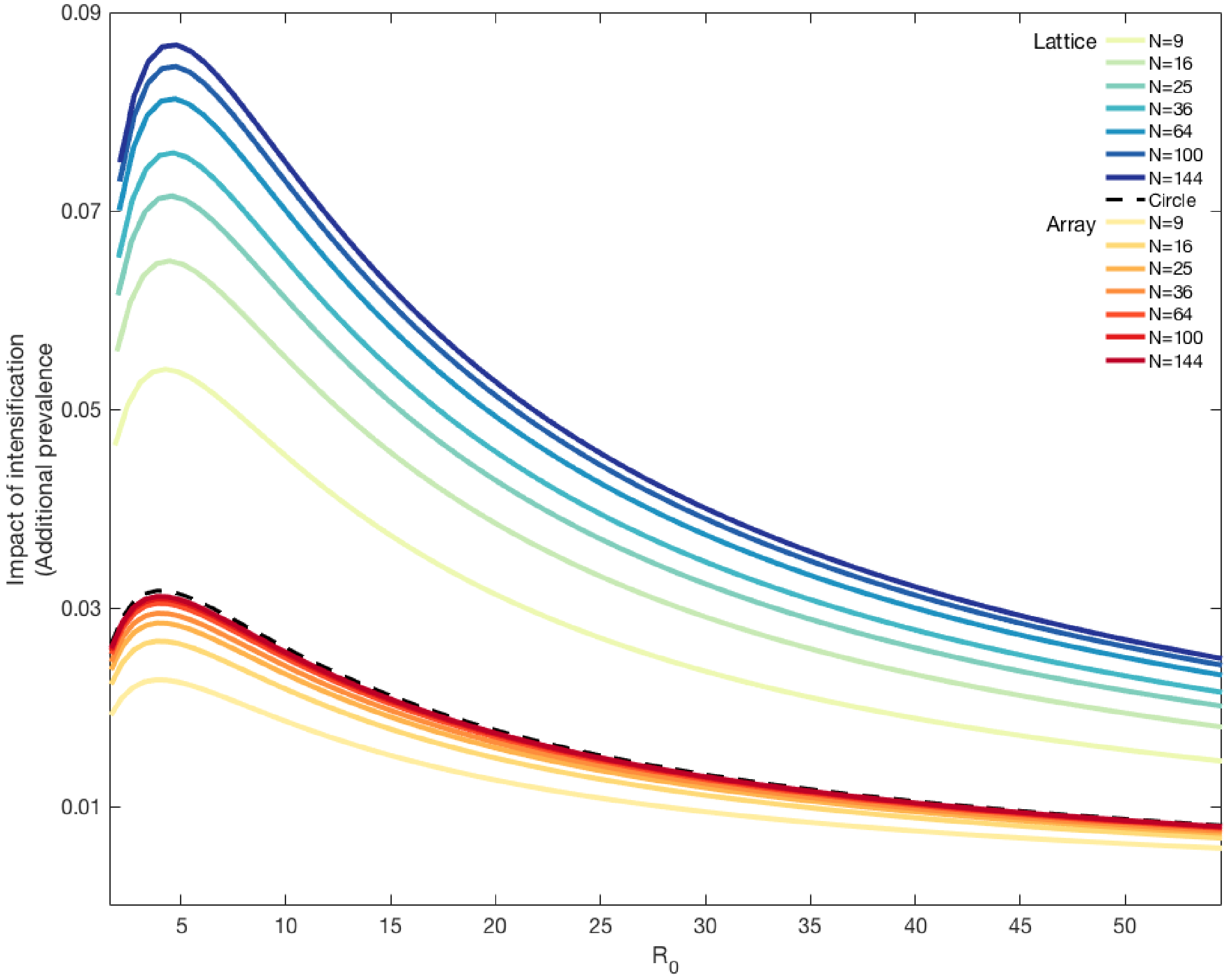

Figure S8. Impact of intensification on disease burden analytically derived from the mathematical model, showing only the effect of increasing apiary size under different spatial configurations; movement rate between colonies is held constant. The circular apiary dynamics are independent of intensification, thus remaining constant through intensification (i.e. increasing n). The impact of intensification for the circular apiary (of any size) is in black.

Supplementary Information Section 4

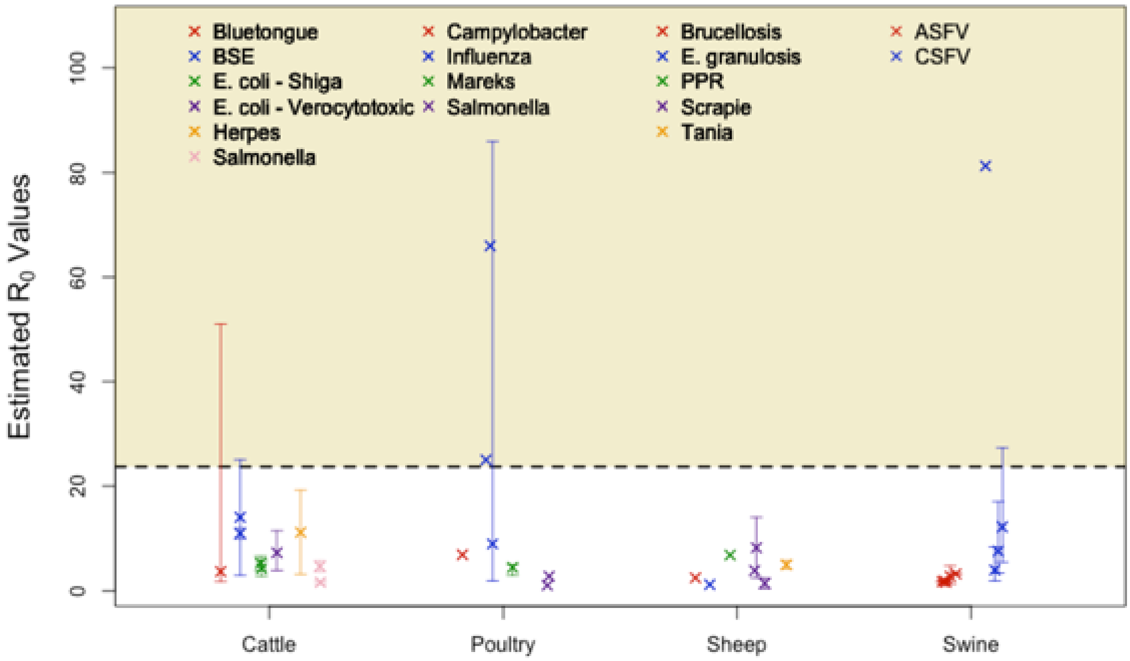

Figure S9 Examples of R_0 values for other agricultural livestock diseases (see Supplementary Information Section 4 for references), spanning a range of different farming stages and practices. This figure is not intended as an exhaustive or representative summary of agricultural disease R_0 values, but represents what is readily available in the literature. We highlight the lower boundary shown in Fig. 5, which is our best estimate of the lower R_0 value for *N. ceranae* in honeybees.

Table S1

Estimates for R_0 values for agricultural livestock diseases available across the literature.

R_0Value	Lower	Upper	Host	Disease	Ref
4.5	3	5	Poultry	Mareks	Atkins et al 2013
3.24	3.21	3.27	Swine	ASFV	Barongo et al 2015
1.64	1.56	1.72	Swine	ASFV	Barongo et al 2015
1.91	1.87	1.94	Swine	ASFV	Barongo et al 2015
1.78	1.74	1.81	Swine	ASFV	Barongo et al 2015
66	66	66	Poultry	Influenza	Bos et al 2007
25	25	25	Poultry	Influenza	Bos et al 2007
5	4.2	5.8	Sheep	Tania	Cabrera et al 1995
1.2	1.2	1.2	Sheep	E. granulosis	Cabrera et al 1995
4	1.9	8.4	Swine	CSFV	Durand et al 2009
7.6	3.4	17	Swine	CSFV	Durand et al 2009
12.2	5.5	27.3	Swine	CSFV	Durand et al 2009
11	10	12	Cattle	BSE	Ferguson et al 1999
2.8	1.3	4.8	Swine	ASFV	Guinat et al 2016
18	6.9	46.9	Swine	ASFV	Sanches-Vizcaino et al 2015
9.8	3.9	15.6	Swine	ASFV	Gulenkin et al 2011
8.25	2.5	14	Sheep	Scrapie	Hagenaars et al 2003
2.5	2.5	2.5	Sheep	Brucellosis	Hou et al 2013
14	3	25	Cattle	BSE	Koeijer et al 2004
4.3	2.8	5.9	Cattle	E. coli - Shiga	Laegried and Keen 2004
5.3	3.9	6.6	Cattle	E. coli - Shiga	Laegried and Keen 2004
1.62	1.34	1.9	Cattle	Salmonella	Lanzas et al 2008
4.7	3.65	5.75	Cattle	Salmonella	Lanzas et al 2008
81.3	81.3	81.3	Swine	CSFV	Leavens et al 1998
3.9	3.9	3.9	Sheep	Scrapie	Matthews et al 1999
11.2	3.2	19.2	Cattle	Herpes	Mollema et al 2005
2.8	2.8	2.8	Poultry	Salmonella	Rabsch et al 2000
1.05	1.05	1.05	Poultry	Salmonella	Rabsch et al 2000
3.7	1.8	51	Cattle	Bluetongue	Santman-Berends et al 2013
7.3	3.92	11.51	Cattle	E. coli - Verocytotoxic	Schouten et al 2009
1.43	0.42	2.43	Sheep	Scrapie	Truscott and Ferguson 2009
6.85	6.85	6.85	Sheep	PPR	Zahur et al 2009
9	1.9	86	Poultry	Influenza	Bouma et al 2009
6.92	6.92	6.92	Poultry	Campylobacter	Goddard et al 2014

References – Section 4

Atkins, K.E., Read, A.F., Savill, N.J., Renz, K.G., Islam, A.F., Walkden-Brown, S.W. & Woolhouse, M.E.J. (2013) Vaccination and Reduced Cohort Duration Can Drive Virulence Evolution: Marek’s Disease Virus and Industrialized Agriculture. *Evolution*, **67**, 851–860.

Barongo, M.B., Ståhl, K., Bett, B., Bishop, R.P., Fèvre, E.M., Aliro, T., Okoth, E., Masembe, C., Knobel, D. & Ssematimba, A. (2015) Estimating the Basic Reproductive Number (R0) for African Swine Fever Virus (ASFV) Transmission between Pig Herds in Uganda. *PLOS ONE*, **10**, e0125842.

Bos, M.E.H., Boven, M.V., Nielen, M., Bouma, A., Elbers, A.R.W., Nodelijk, G., Koch, G., Stegeman, A. & Jong, M.C.M.D. (2007) Estimating the day of highly pathogenic avian influenza (H7N7) virus introduction into a poultry flock based on mortality data. *Veterinary Research*, **38**, 493–504.

Bouma, A., Claassen, I., Natih, K., Klinkenberg, D., Donnelly, C.A., Koch, G. & Boven, M. van. (2009) Estimation of Transmission Parameters of H5N1 Avian Influenza Virus in Chickens. *PLOS Pathogens*, **5**, e1000281.

Cabrera, P.A., Haran, G., Benavidez, U., Valledor, S., Perera, G., Lloyd, S., Gemmell, M.A., Baraibar, M., Morana, A., Maissonave, J. & Carballo, M. (1995) Transmission dynamics of Echinococcus granulosus, Taenia hydatigena and Taenia ovis in sheep in uruguay. *International Journal for Parasitology*, **25**, 807–813.

Durand, B., Davila, S., Cariolet, R., Mesplède, A. & Le Potier, M.-F. (2009) Comparison of viraemia- and clinical-based estimates of within- and between-pen transmission of classical swine fever virus from three transmission experiments. *Veterinary Microbiology*, **135**, 196–204.

Ferguson, N.M., Donnelly, C.A., Woolhouse, M.E.J. & Anderson, R.M. (1999) Estimation of the basic reproduction number of BSE: the intensity of transmission in British cattle. *Proceedings of the Royal Society of London B: Biological Sciences*, **266**, 23–32.

Goddard, A.D., Arnold, M.E., Allen, V.M. & Snary, E.L. (2014) Estimating the time at which commercial broiler flocks in Great Britain become infected with Campylobacter: a Bayesian approach. *Epidemiology & Infection*, **142**, 1884–1892.

Guinat, C., Gubbins, S., Vergne, T., Gonzales, J.L., Dixon, L. & Pfeiffer, D.U. (2016) Experimental pig-to-pig transmission dynamics for African swine fever virus, Georgia 2007/1 strain. *Epidemiology & Infection*, **144**, 25–34.

Gulenkin, V.M., Korennoy, F.I., Karaulov, A.K. & Dudnikov, S.A. (2011) Cartographical analysis of African swine fever outbreaks in the territory of the Russian Federation and computer modeling of the basic reproduction ratio. *Preventive Veterinary Medicine*, **102**, 167–174.

Hagenaars, T.J., Donnelly, C.A., Ferguson, N.M. & Anderson, R.M. (2003) Dynamics of a scrapie outbreak in a flock of Romanov sheep – estimation of transmission parameters. *Epidemiology & Infection*, **131**, 1015–1022.

Hou, Q., Sun, X., Zhang, J., Liu, Y., Wang, Y. & Jin, Z. (2013) Modeling the transmission dynamics of sheep brucellosis in Inner Mongolia Autonomous Region, China. *Mathematical Biosciences*, **242**, 51–58.

Koeijer, A. de, Heesterbeek, H., Schreuder, B., Oberthür, R., Wilesmith, J., Roermund, H. van & Jong, M. de. (2004) Quantifying BSE control by calculating the basic reproduction ratio R0 for the infection among cattle. *Journal of Mathematical Biology*, **48**, 1–22.

Laegreid, W.W. & Keen, J.E. (2004) Estimation of the basic reproduction ratio (R0) for Shiga toxin-producing Escherichia coli O157:H7 (STEC O157) in beef calves. *Epidemiology & Infection*, **132**, 291–295.

Lanzas, C., Brien, S., Ivanek, R., Lo, Y., Chapagain, P.P., Ray, K.A., Ayscue, P., Warnick, L.D. & Gröhn, Y.T. (2008) The effect of heterogeneous infectious period and contagiousness on the dynamics of Salmonella transmission in dairy cattle. *Epidemiology & Infection*, **136**, 1496–1510.

Leavens, H., Koenen, F., Deluyker, H., Berkvens, D. & Kruif, A. de. (1998) An experimental infection with classical swine fever virus in weaner pigs. *Veterinary Quarterly*, **20**, 41–45.

Matthews, L., Woolhouse, M.E.J. & Hunter, N. (1999) The basic reproduction number for scrapie. *Proceedings of the Royal Society of London B: Biological Sciences*, **266**, 1085–1090.

Mollema, L., Jong, M.C.M.D. & Boven, M.V. (2005) Prolonged persistence of bovine herpesvirus in small cattle herds: a model-based analysis. *Epidemiology & Infection*, **133**, 137–148.

Rabsch, W., Hargis, B.M., Tsolis, R.M., Kingsley, R.A., Hinz, K.H., Tschäpe, H. & Bäumler, A.J. (2000) Competitive exclusion of Salmonella enteritidis by Salmonella gallinarum in poultry. *Emerging Infectious Diseases*, **6**, 443–448.

Sánchez-Vizcaíno, J.M., Mur, L., Gomez-Villamandos, J.C. & Carrasco, L. (2015) An Update on the Epidemiology and Pathology of African Swine Fever. *Journal of Comparative Pathology*, **152**, 9–21.

Santman-Berends, I.M.G.A., Stegeman, J.A., Vellema, P. & van Schaik, G. (2013) Estimation of the reproduction ratio (R0) of bluetongue based on serological field data and comparison with other BTV transmission models. *Preventive Veterinary Medicine*, **108**, 276–284.

Schouten, J.M., Graat, E. a. M., Frankena, K., Zijderveld, F.V. & Jong, M.C.M.D. (2009) Transmission and quantification of verocytotoxin-producing Escherichia coli O157 in dairy cattle and calves. *Epidemiology & Infection*, **137**, 114–123.

Truscott, J.E. & Ferguson, N.M. (2009) Control of scrapie in the UK sheep population. *Epidemiology & Infection*, **137**, 775–786.

Zahur, A.B., Ullah, A., Irshad, H., Farooq, M.S., Hussain, M., Jahangir, M. & others. (2009) Epidemiological investigations of a peste des petits ruminants (PPR) outbreak in Afghan sheep in Pakistan. *Pakistan Veterinary Journal*, **29**, 174–178.
